# The role of surgery on primary site in metastatic upper urinary tract urothelial carcinoma and a nomogram for predicting the survival of patients with metastatic upper urinary tract urothelial carcinoma

**DOI:** 10.1002/cam4.4327

**Published:** 2021-10-14

**Authors:** Xiaodi Zhang, Ping Wang, Kaiyan QI, Qiao Qiao, Yuanjun Jiang

**Affiliations:** ^1^ Radiation Oncology the First Hospital of China Medical University Shenyang China; ^2^ Department of Radiation Oncology the First Hospital of China Medical University Shenyang China; ^3^ Department of Urology the First Hospital of China Medical University Shenyang, Liaoning China

**Keywords:** metastasis, nomogram, SEER, surgical therapy, urothelial

## Abstract

Metastatic upper urinary tract urothelial carcinoma (mUTUC) is a relatively rare urothelial carcinoma, and little attention has been given to it. Our study established a nomogram by analyzing the prognostic factors of mUTUC to predict the survival of patients and revealed that the role of surgery at the primary tumor site. We extracted our data (2010–2016) from the Surveillance, Epidemiology, and End Results (SEER) database, and 628 patients with distant metastasis were identified. Propensity score matching (PSM) was used to balance the clinical variable bias in a 1:1 ratio. After PSM, we enrolled 502 patients in our study cohort. Univariate and multivariate Cox regression analyses and Kaplan–Meier curves showed that T stage, N stage, hepatic metastasis, surgery, and chemotherapy were prognostic factors for mUTUC before and after PSM. Based on the findings, a nomogram was constructed to predict the 12‐month survival of patients with distant metastasis. The analysis of subgroups of T stage, N stage, and different metastatic sites demonstrated that the survival of patients with T1/T2, N0/N1/N2/N3, metastasis including liver, and metastasis including bone could be improved by a combination of surgery and chemotherapy, while for the patients with T3/T4/TX, NX, metastasis including lung, and metastasis including distant lymph nodes, chemotherapy alone was a better choice to improve their overall survival. Radiotherapy has been proven to be useful for patients with N1/N2/N3 stage. We have provided more precise treatment strategies for stage IV patients. Our research fully affirms the role of surgery on primary site in UTUC patients with distant metastasis and the significance of classifying the patients into subgroups by integrating variables including T stage, N stage, and different metastatic sites to select the optimal treatment method.

## Graphical abstract

It is a research about prognostic factors of metastatic upper urinary tract urothelial carcinoma (mUTUC) including metastatic patterns, therapy modes, and T/N stage based on the data extracted from SEER database. It reveals that the role of surgery on primary site is significant in the treatment of metastatic upper urinary tract urothelial carcinoma.

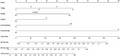

## 1 INTRODUCTION

Urothelial carcinoma (UC) is derived from the lower (bladder and urethra) and upper (renal pelvis and ureter) urinary tracts.^1^ The ureter and renal pelvis are usually combined into upper urinary tract urothelial carcinoma (UTUC). It is an aggressive tumor characterized by invasive growth and high incidence of variant histology. As the most common tumor of the urinary system, bladder tumors account for 90%–95% of UCs, while UTUCs are uncommon and account for only 5%–10% of UCs.^2^ Metastatic UTUCs (mUTUCs) are even rarer, whose diagnosis suggests the median survival duration of only 6 months, accounting for only 10% of UTUCs. Moreover, there has been a prominent increase in the incidence of metastatic UTUC from 0.1 to 0.4 per 100,000 person‐years over the past 30 years.^3^

For patients with high‐risk nonmetastatic UTUC, the standard treatment is open radical nephroureterectomy (RNU) with bladder cuff excision, regardless of tumor location.^4^ However, for patients with metastatic UTUC (mUTUC), RNU is recommended for palliative treatment in the current guidelines and platinum‐based combination chemotherapy––especially using cisplatin––has taken a central role in the management of mUTUC for a long while. However, not all patients can receive chemotherapy because of their impaired renal function and chemotherapy‐related toxicity, particularly nephrotoxicity due to platinum derivatives, which may also significantly reduce the survival of the patients.^5^ This means that managing the patients with mUTUC can cause a dilemma in the clinic. In recent years some observational studies have revealed that surgery on primary site may not be without an impact on mUTUC outcomes, but the conclusions have been limited by small patient numbers and inhomogeneity of the study populations as related to patient selection, pathologic evaluation, and treatment.^4^, ^6^, ^7^, ^8^, ^9^, ^10^, ^11^, ^12^, ^13^ Besides, very few studies have been performed on a model that can predict the prognosis of patients with metastatic UTUCs. Consequently, it is necessary to clarify the role of surgery in patients with mUTUC and determine the independent prognostic factors, which were used to establish a model to predict the survival of the patients (at 4, 8, and 12 months). Finally, through further stratified analysis, a better treatment strategy for stage IV patients with different T/N stages and patients with different metastatic sites was revealed.

## 2 MATERIALS AND METHODS

### 2.1 Study cohort

We carried out a retrospective study using data from the Surveillance, Epidemiology, and End Results (SEER) database. Our inclusion criteria were as follows: (1) patients were diagnosed with neoplasms of the renal pelvis and ureter between 2010 and 2016 and (2) carcinoma of the renal pelvis and ureter was their first primary malignancy. The exclusion criteria included were as follows: the information about age at diagnosis, race, sex, histologic type, grade, T stage, N stage, M stage, surgery on primary site, surgery for regional lymph nodes, radiotherapy, chemotherapy, the scope of regional lymph node surgery, metastatic information, survival months, and current status were unavailable. It is worth mentioning that the surgery group included patients with surgery at the primary site for codes 30–80, including partial or subtotal nephrectomy (kidney or renal pelvis) or partial ureterectomy (ureter) (e.g., segmental resection, wedge resection), complete/total/simple nephrectomy (kidney parenchyma) or nephroureterectomy (including the bladder cuff for the renal pelvis or ureter), radical nephrectomy (including removal of a portion of the vena cava, the adrenal gland(s), Gerota's fascia, perinephric fat, or partial/total ureter), and any nephrectomy in continuity with the resection of other organ(s) (colon, bladder).

### 2.2 Variable definitions and study endpoints

The variables extracted from the SEER database contained age at diagnosis, race, sex, pure or variant histology, grade, the AJCC Staging Manual tumor stage (7^th^ edition) including TX/T0/Ta/Tis/T1/T2/T3/T4/NX/N0/N1/N2/N3/M0/M1, metastatic information, and treatment modalities including surgery on primary site, surgery on the regional lymph nodes, radiotherapy, chemotherapy, and the scope of regional lymph nodes surgery. Metastatic information included the distant metastatic sites that were identified at the time of diagnosis and the number of metastatic sites. The study endpoint was the overall survival (OS). According to the European Association of Urology Guidelines on Upper Urinary Tract Urothelial Cell Carcinoma (2020 Update), upper urinary tract tumors with variant histology (UTVH) are one of the high‐risk factors.^6^ Consequently, the patients in our study were divided into two groups by their histology: one group was UTVH including squamous cell carcinoma (SCC), adenocarcinoma, neuroendocrine carcinoma, and other kinds of UTVH^15^ and the other was pure upper urinary tract urothelial cell carcinoma (PUC).

### 2.3 Statistical analysis

We used the Kaplan–Meier methods and the log‐rank tests to compare the survival times. Univariate and multivariate Cox regression analyses revealed the independent prognostic variables related to OS. Before the univariate and multivariate Cox regression analyses, a proportional hazards assumption (PH Assumption) was performed to filter the variables that could be included in the regression model. We set a 1:1 ratio to reduce bias by the ‘MatchIt’ R package using a propensity score matching (PSM) method. The chi‐squared test and the Fisher's exact test were used to make comparisons for categorical variables. A *p* value <0.05 was considered statistically significant in all analyses. We used R version 4.0.3, IBM SPSS Statistics software version 24, and GraphPad Prism version 8 to perform all statistical analyses.

## 3 RESULTS

### 3.1 Patient characteristics and a poor prognosis predicted by distant metastasis

We enrolled 6724 patients diagnosed with upper urinary tract urothelial carcinoma. Among the final cohort, 628 (9.34%) were recorded as having distant metastasis at the time of diagnosis. The demographic and clinicopathological characteristics of the metastatic group and nonmetastatic group are shown in Table S1. Compared to the nonmetastatic group, the metastatic group tended to have a higher T stage (T4/TX), a higher N stage (N1, N2, N3, NX), and a higher grade (poorly differentiated; Grade III). From the perspective of treatment, distant metastatic patients had a higher probability of receiving chemotherapy and radiotherapy instead of surgery at the primary site. Additionally, it was less likely for them to choose surgery of regional lymph nodes in favor of biopsy in comparison with those without distant metastasis.

**FIGURE 1:**
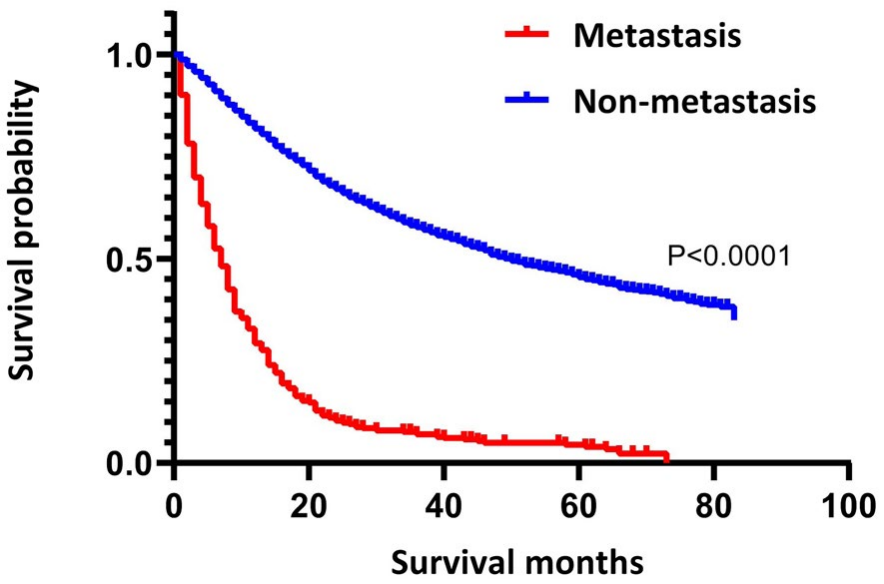
Kaplan–Meier survival curves for upper urinary tract urothelial carcinoma with and without distant metastasis (*p* < 0.0001)

Furthermore, univariate and multivariate Cox regression analyses showed that distant metastasis was an independent prognostic factor for OS (Table S2). The K–M survival analysis also showed that the survival of patients with distant metastasis was much worse than that of patients without distant metastasis (*p* < 0.0001, Figure 1). Then, we performed the univariate and multivariate Cox regression analyses on the metastatic group, which revealed that T stage, metastasis including liver, surgery, and chemotherapy were independent prognostic factors for mUTUC (Table S3).

### 3.2 Metastatic patterns and prognostic analysis

#### 3.2.1 The sites of distant metastasis

**FIGURE 2:**
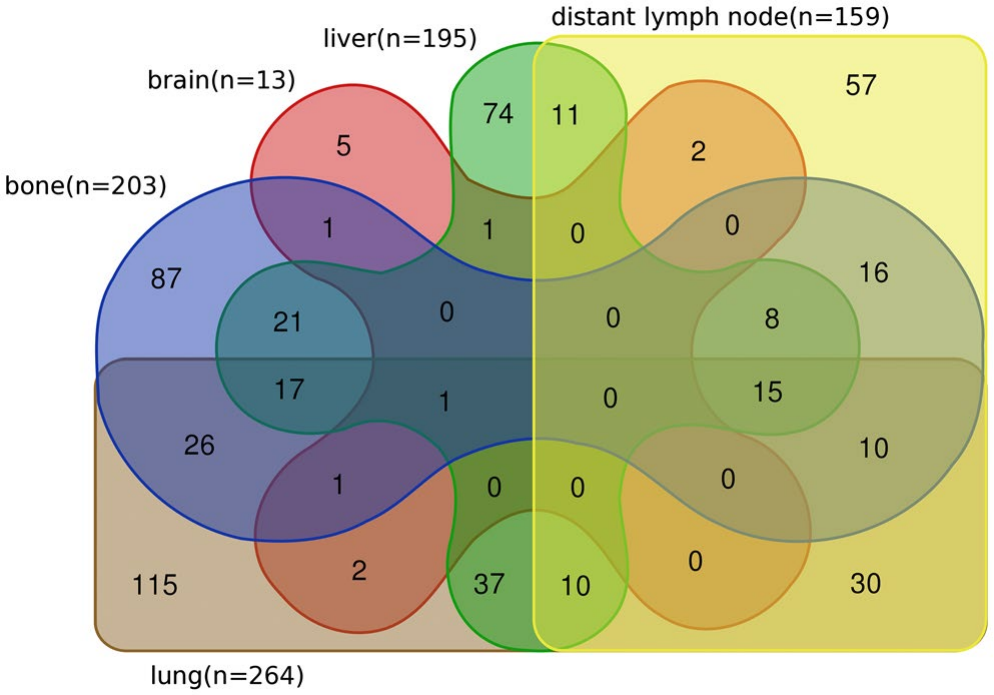
Venn diagram of the distributions of distant metastatic organs in metastatic upper tract urothelial carcinoma patients

**FIGURE 3:**
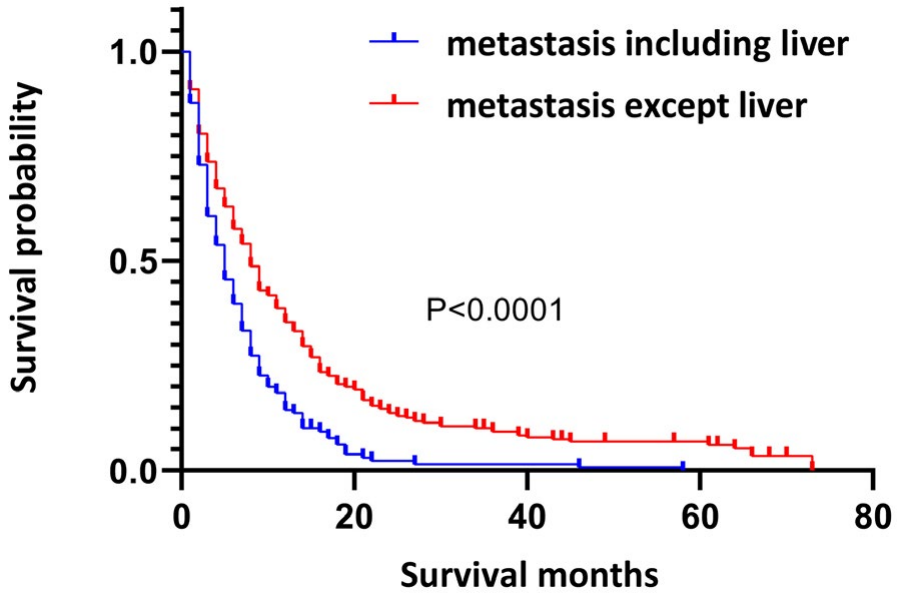
Kaplan–Meier survival curves for metastatic upper urinary tract urothelial carcinoma patients with and without liver metastasis (*p* < 0.0001)

Lung metastasis was the most common site (264, 42.04%), followed by bone (203, 32.32%), liver (195, 31.05%), distant lymph nodes (159, 25.32%), and brain (13, 0.16%), but the brain was not included in this study because the sample was too small (Figure 2). In the remaining four sites, the Kaplan–Meier curves demonstrated that the sites of distant metastasis were associated with the prognosis (*p* = 0.0136, Figure S1A). Patients with liver metastasis had a worse prognosis than those without liver metastasis (*p* < 0.0001, Figure 3), but no detectable difference was found in other three sites (Figure S1B–D). Kaplan–Meier analysis was also performed on metastatic patients with only one site. The prognosis of liver‐only metastasis was worse than that of other one‐site metastases (*p* < 0.0001, Figure S1E), and no detectable difference was found among the other three sites (Figure S1F–H). It follows that liver metastasis was closely correlated with a worse OS.

#### 3.2.2 The number of distant metastasis

The most common was one site (339, 53.98%), followed by two sites (147, 23.41%), three sites (45, 7.17%), and four sites (81, 12.90%). Figure 2 shows the details of the distribution of distant metastatic numbers.

**FIGURE 4:**
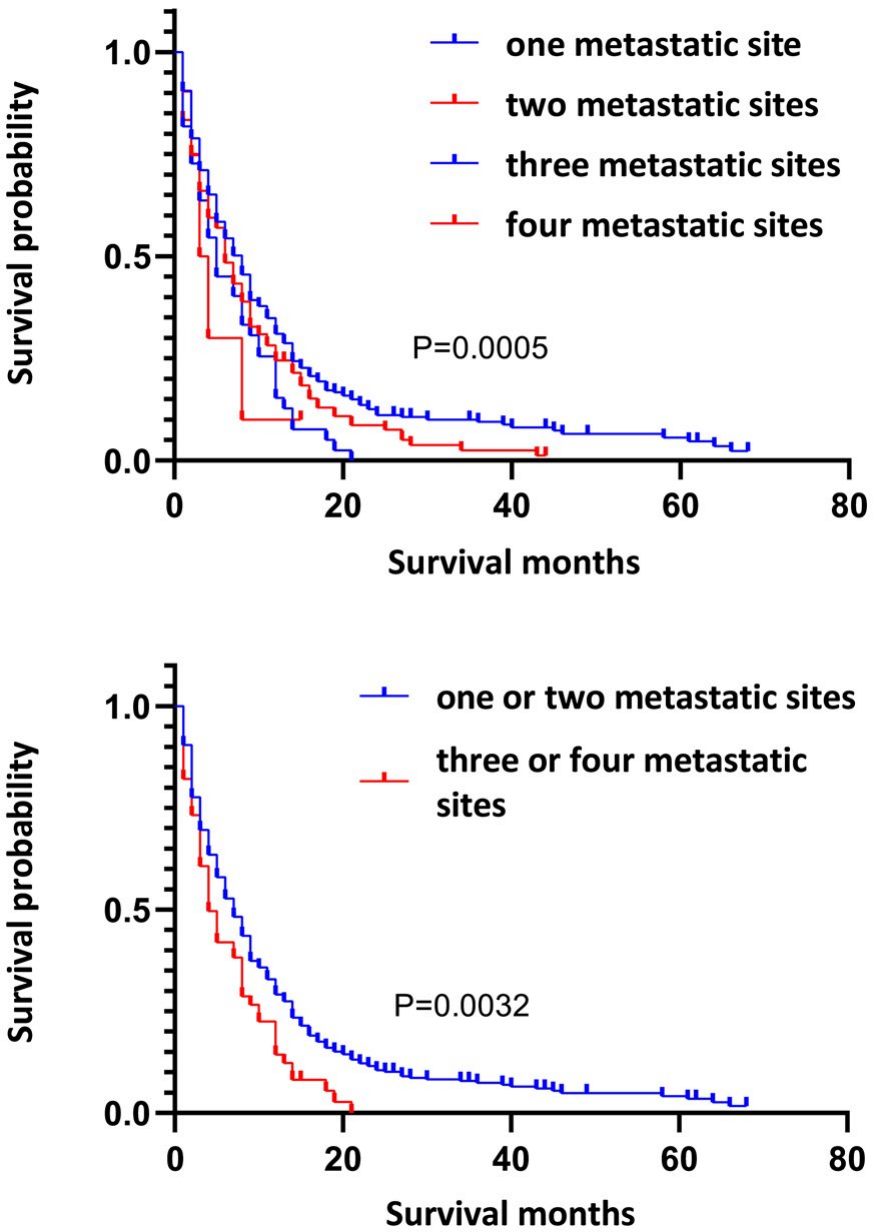
(A) Kaplan–Meier survival curves for metastatic upper urinary tract urothelial carcinoma with one, two, three, or four sites of metastasis (*p* = 0.0005). (b). Kaplan–Meier survival curves for metastatic upper urinary tract urothelial carcinoma with one or two and more than two sites of metastasis (*p* = 0.0032)

Through Kaplan–Meier curves, we found that OS l of patients varied with the number of metastatic sites (*p* = 0.0005, Figure 4A). Then, we had a further analysis to patients with different numbers of metastatic sites. In all patients with distant metastasis, the OS with a single metastatic site was not significantly different from that of two metastatic sites (Figure S2A), but it was better than that with three or four metastatic sites (*p* = 0.0068, *p* = 0.0443, Figure S2B,C). Moreover, there was no difference in prognosis between those with three and four metastatic sites (Figure S2D). Our K–M survival curves confirmed that the prognosis of patients with more than two sites of metastasis was much worse than those with one or two sites (*p* = 0.0032, Figure 4B).

### 3.3 The role of surgery at the primary site in mUTUC after PSM and the nomogram established from the prognostic factors

To more accurately evaluate the effect of surgery at the primary site in metastatic cancers of the renal pelvis and ureter, PSM was used to unify the background between the surgery group and non‐surgery group in a 1:1 ratio, and 502 patients were eventually enrolled in our study. After PSM, the difference in age at diagnosis (years), metastasis including bone, metastasis including liver, and the number of metastatic sites were balanced, while the differences in grade, T stage, and the scope of regional lymph node surgery were still significant. The clinicopathological features of the two groups before and after PSM are shown in Table 1.

**TABLE 1:** Clinicopathological features of metastatic upper urinary tract urothelial carcinoma patients with and without surgery before and after propensity score matching

	Before PSM			After PSM		
	No surgery (*N* = 309)	Surgery (*N* = 319)	*p* value	No surgery (*N* = 251)	Surgery (*N* = 251)	*p* value
Age at diagnosis (years)			0.022			0.955
<70	109 (35.3%)	141 (44.2%)		95 (38.0%)	96 (38.2%)	
≥70	200 (64.7%)	178 (55.8%)		155 (32.0%)	155 (31.8%)	
Race			0.899			0.907
Black	18 (5.8%)	16 (5.0%)		13 (5.2%)	11 (4.4%)	
White	28 (9.1%)	30 (9.4%)		22 (8.8%)	23 (9.2%)	
Other	263 (85.1%)	273 (85.6%)		215 (86.0%)	217 (86.5%)	
Histological type			0.329			0.317
PUC	284 (91.9%)	286 (89.7%)		227 (90.8%)	221 (88.0%)	
UTVH	25 (8.1%)	33 (10.3%)		23 (9.2%)	30 (12.0%)	
Grade			<0.0001			<0.0001
I	6 (1.9%)	2 (0.6%)		5 (2.0%)	2 (0.8%)	
II	27 (8.7%)	15 (4.7%)		20 (8.0%)	10 (4.0%)	
III	123 (39.8%)	86 (27.0%)		104 (41.6%)	63 (25.1%)	
IV	153 (49.5%)	216 (67.7%)		121 (48.4%)	176 (70.1%)	
T stage			<0.0001			<0.0001
T1	75 (24.3%)	10 (3.1%)		48 (19.2%)	7 (2.8%)	
T2	23 (7.4%)	14 (4.4%)		19 (7.6%)	(5.2%)	
T3	42 (13.6%)	154 (48.3%)		35 (14.0%)	112 (44.6%)	
T4	58 (18.8%)	139 (43.6%)		48 (19.2%)	117 (46.6%)	
TX	111 (35.9%)	2 (0.6%)		100 (40.0%)	2 (0.8%)	
N stage			0.114			0.086
N0	38 (12.3%)	24 (7.5%)		35 (14.0%)	20 (8.0%)	
N1/N2/N3	103 (33.3%)	119 (37.3%)		78 (31.2%)	89 (35.5%)	
NX	168 (54.4%)	176 (55.2%)		137 (54.8%)	142 (56.6%)	
Scope of regional lymph nodes surgery			<0.0001			<0.0001
No surgery	300 (97.1%)	151 (47.3%)		242 (96.8%)	127 (50.6%)	
Only biopsy	7 (2.3%)	5 (1.6%)		6 (2.4%)	5 (2.0%)	
Surgery but no LN removed	2 (0.6%)	163 (51.1%)		2 (0.8%)	119 (47.4%)	
Radiotherapy			0.620			0.396
No/unknown	264 (85.4%)	268 (84.0%)		215 (86.0%)	209 (83.3%)	
Yes	45 (14.6%)	51 (16.0%)		35 (14.0%)	42 (16.7%)	
Chemotherapy			0.614			0.447
No/unknown	142 (46.0%)	153 (48.0%)		117 (46.8%)	126 (50.2%)	
Yes	167 (54.0%)	166 (52.0%)		133 (53.2%)	125 (49.8%)	
Metastasis including bone			0.016			0.615
No	195 (63.1%)	230 (72.1%)		162 (64.8%)	168 (66.9%)	
Yes	114 (36.9%)	89 (27.9%)		88 (35.2%)	83 (33.1%)	
Metastasis including brain			0.144			0.339
No	300 (97.1%)	315 (98.7%)		244 (97.6%)	248 (98.8%)	
Yes	9 (2.9%)	4 (1.3%)		6 (2.4%)	3 (1.2%)	
Metastasis including liver			0.015			0.399
No	199 (64.4%)	234 (73.4%)		178 (71.2%)	170 (67.7%)	
Yes	110 (35.6%)	85 (26.6%)		72 (28.8%)	81 (32.3%)	
Metastasis including lung			0.190			0.689
No	171 (55.3%)	193 (60.5%)		143 (57.2%)	148 (59.0%)	
Yes	138 (44.7%)	126 (39.5%)		107 (42.8%)	103 (41.0%)	
Metastasis including distant lymph node			0.154			0.665
No	223 (72.2%)	246 (77.1%)		182 (72.8%)	187 (74.5%)	
Yes	86 (27.8%)	73 (22.9%)		68 (27.2%)	64 (25.5%)	

Abbreviations: PUC, pure upper urinary tract urothelial cell carcinoma; UTVH, upper urinary tract tumors with variant histology.

**FIGURE 5:**
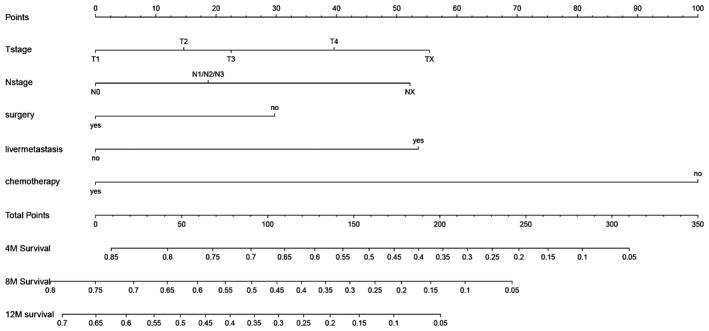
Nomogram predicting 4, 8, and 12‐months overall survival in metastatic upper urinary tract urothelial carcinoma with distant metastasis (4 M survival: 4‐month overall survival; 8 M survival: 8‐month overall survival; and 12 M survival: 12‐month overall survival)

**FIGURE 6:**
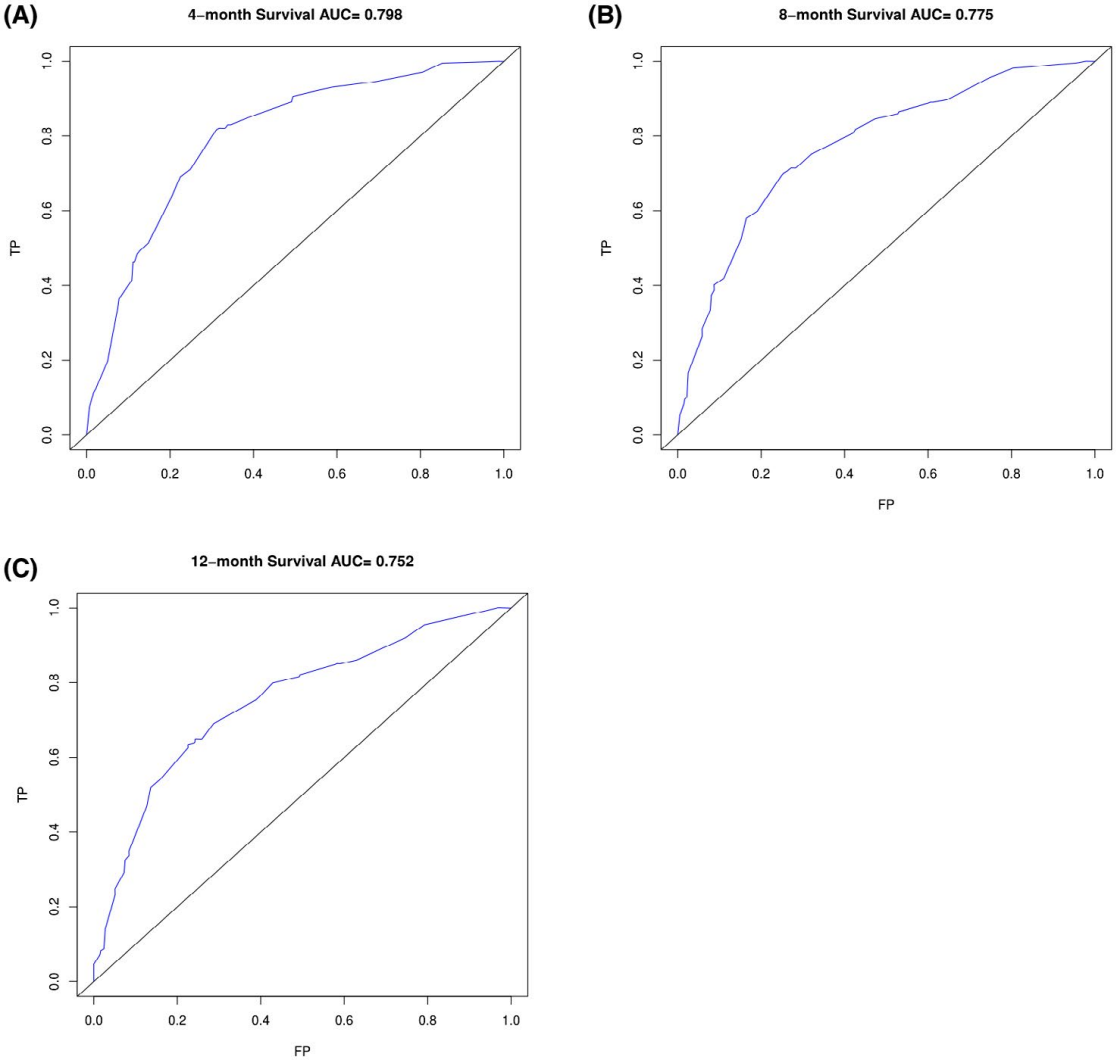
ROC curves of 4, 8, and 12‐months overall survival in metastatic upper urinary tract urothelial carcinoma with distant metastasis (A. ROC curve of 4‐month survival, AUC = 0.798; B. ROC curve of 8‐month survival, AUC = 0.775; and C. ROC curve of 12‐month survival, AUC = 0.752)

**FIGURE 7:**
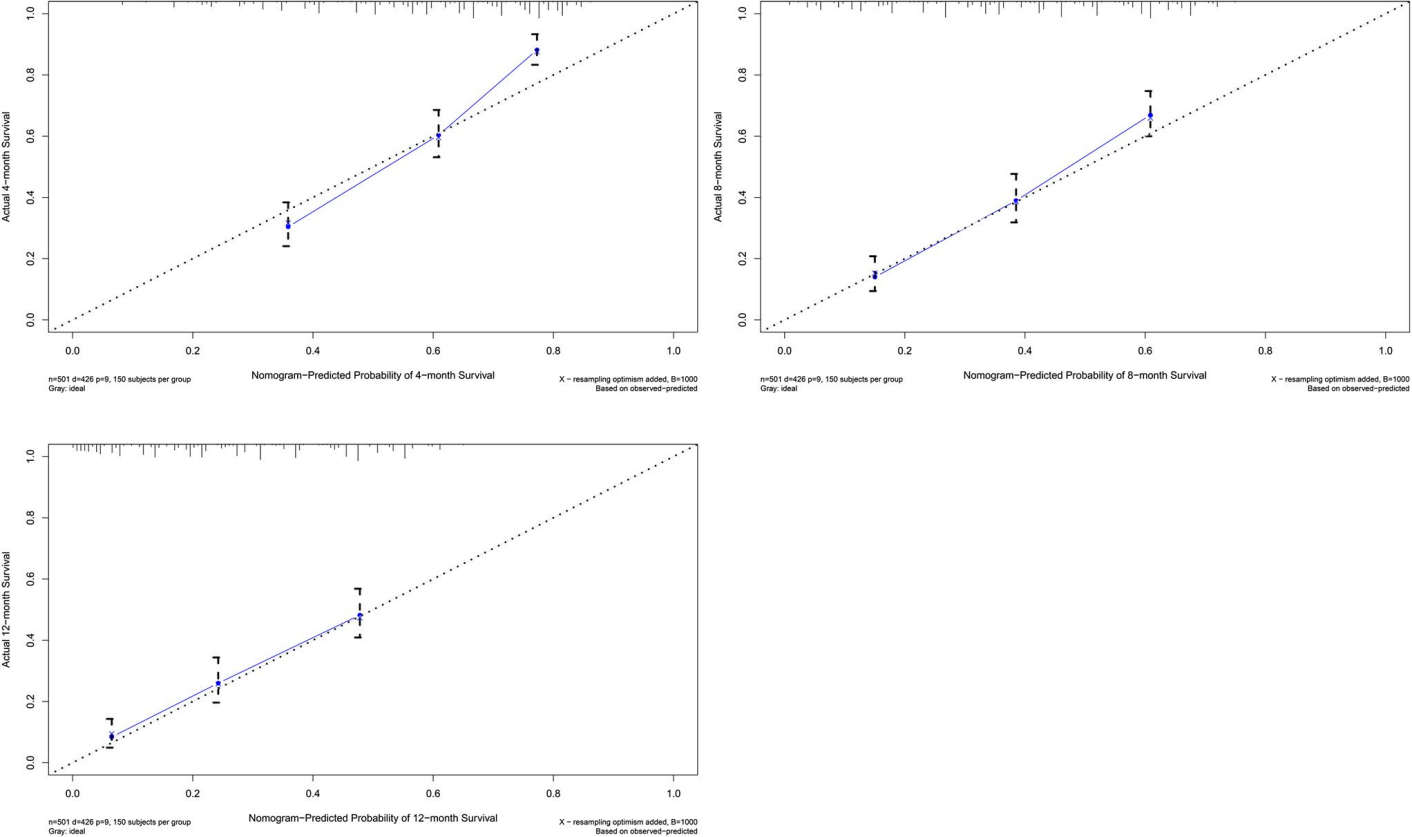
Calibration plots for the nomogram to predict the probability of 4, 8, and 12‐months survival of metastatic upper urinary tract urothelial carcinoma (A. calibration plots for 4‐month survival; B. calibration plots for 8‐month survival; and C. calibration plots for 12‐month survival)

Univariate and multivariate Cox regression analyses suggested that OS was associated with T stage, N stage, metastasis including liver, and treatment patterns (surgery and chemotherapy) after PSM (Table 2). According to these five independent prognostic factors, a nomogram was established to predict the 12‐month survival of patients with distant metastasis (Figure 5). The AUC of this nomogram for predicting 4, 8, and 12‐months OS was 0.798, 0.775, and 0.752, respectively (Figure 6A–C). We also verified the superior accuracy of this model by calibration plots, as shown (Figure 7A–C).

**TABLE 2:** Univariable and multivariable Cox regression model analyses of overall survival of 502 patients with metastatic upper urinary tract urothelial carcinoma after PSM

Variables	Level	Univariable			Multivariable		
		*p* value	HR	95% CI	*p* value	HR	95% CI
Age at diagnosis (years)	70–79	0.064					
	>79	0.064	1.205	0.989–1.468			
Race	Black (ref)	0.680					
	White	0.657	0.883	0.510–1.529			
	Other	0.418	0.826	0.520–1.312			
Histologic type	PUC (ref)	0.826					
	UTVH	0.826	1.035	0.764–1.402			
Grade	I (ref)	0.183					
	II	0.145	2.206	0.762–6.383			
	III	0.045	2.771	1.022–7.513			
	IV	0.057	2.622	0.972–7.074			
T stage	T1 (ref)	0.001			0.037		
	T2	0.696	1.102	0.677–1.793	0.373	1.249	0.765–2.040
	T3	0.906	1.022	0.718–1.454	0.454	1.155	0.793–1.682
	T4	0.074	1.369	0.970–1.934	0.043	1.453	1.012–2.085
	TX	0.003	1.758	1.211–2.553	0.009	1.657	1.133–2.421
N stage	N0 (ref)	0.021			0.021		
	N1/N2/N3	0.035	1.706	1.511–1.976	0.006	0.630	1.453–1.875
	NX	0.006	1.647	1.476–1.880	0.061	0.743	1.544–2.014
Radiotherapy	No/unknown	0.043					
	Yes	0.043	0.758	0.579–0.991			
Chemotherapy	No (ref)	<0.0001			<0.0001		
	Yes	<0.0001	0.427	0.351–0.519	<0.0001	0.400	0.327–0.491
Surgery	No (ref)	0.004			0.021		
	Yes	0.004	0.758	0.626–0.917	0.021	0.758	0.598–0.960
Surgery about regional lymph nodes	No surgery (ref)	0.163					
	Only biopsy	0.830	0.930	0.479–1.805			
	Surgery and lymph node removed	0.057	0.802	0.640–1.006			
Metastatic including bone	No (ref)	0.442					
	Yes	0.442	1.082	0.885–1.323			
Metastatic including liver	No (ref)	<0.0001			<0.0001		
	Yes	<0.0001	1.687	1.372–2.075	<0.0001	1.628	.1316–2.013
Metastatic including lung	No (ref)	0.157					
	Yes	0.157	1.150	0.948–1.395			
Metastatic including distant lymph node	No (ref)	0.125					
	Yes	0.125	0.840	0.673–1.050			
The number of metastatic sites	One or two sites (ref)	0.010					
	Three or four sites	0.036	1.401	1.023–1.919			
	Distant metastatic sites cannot be assessed	0.002	1.914	1.257–2.913			

Abbreviations: PUC, pure upper urinary tract urothelial cell carcinoma; UTVH, upper urinary tract tumors with variant histology.

### 3.4 **Stratified OS analysis of T stage after PSM**.

Because the sample sizes of T1 and T2 were both relatively small and the Kaplan–Meier analysis suggested no diversity of OS between them (Figure S3), we combined T1 and T2 into one group to perform univariate and multivariate Cox analyses, which showed that surgery, chemotherapy, and metastasis including liver were independent prognostic features in T1/2 stage patients (Table S4). Combining surgery with chemotherapy to treat metastatic cancers significantly improved OS compared with the other therapy modes (*p* = 0.0430, Figure S4). In the T3 and TX groups, patients with metastasis including liver or those without chemotherapy, had a much worse prognosis than those without liver metastasis or those undergoing chemotherapy (Tables S5 and S7), while chemotherapy was the only method used to treat patients with T4 (Table S6). As described above, patients with higher T stages could only benefit from chemotherapy instead of surgery and chemotherapy, the same as in the T1/2 stage group.

### 3.5 **Stratified OS analysis of N stage after PSM**.

Univariate and multivariate Cox analyses showed that surgery, chemotherapy, and histological types were independent prognostic factors in N0 stage patients (Table S8). Furthermore, Kaplan–Meier survival analysis demonstrated that the combination of surgery and chemotherapy to treat metastatic cancers could improve the prognosis significantly compared with other therapy modes (*p *< 0.0001, Figure S5). In addition, in terms of histological types, the OS of UTVH decreased distinctly compared with that of PUC (*p *= 0.0027, Figure S6). In the N1/N2/N3 group, T stage, metastasis including liver, and therapy mode containing surgery, chemotherapy, and radiotherapy were independent prognostic factors of these patients (Table S9). Kaplan–Meier curves revealed that radiotherapy could improve the prognosis of these patients (*p* = 0.0196, Figure S7), and in terms of surgery and chemotherapy, the combination of them suggested a better prognosis compared to surgery alone, chemotherapy alone, and no therapy (*p *< 0.0001, Figure S8). According to the comprehensive K–M analysis of these three methods, the prognosis of patients given a combination of surgery, chemotherapy, and radiotherapy was better than that with surgery alone, chemotherapy alone, and radiotherapy alone (*p *= 0.0079, Figure S9). For patients with NX, the only independent prognostic factor to show a benefit was chemotherapy (Table S10).

### 3.6 **Stratified OS analysis of different metastatic sites after PSM**.

First, we performed univariate and multivariate Cox analyses on the liver metastasis group, which indicated that prognostic indicators for this group were surgery and chemotherapy (Table S11). The Kaplan–Meier curves further revealed that combination of surgery and chemotherapy was beneficial to the OS of patients with liver metastasis (*p *< 0.0001, Figure S10). In patients with bone metastasis, surgery, chemotherapy, and the number of metastatic sites influenced OS (Table S12). Similar to liver metastasis, surgery combined with chemotherapy was the best choice for these patients (*p *< 0.0001, Figure S11).

For the metastasis including lung group, chemotherapy and liver metastasis were clearly related to OS (Table S13). And for the metastasis including distant lymph node group, chemotherapy, T stage, and metastasis including bone were related to OS (Table S14).

### 3.7 **Survival analysis of treatment modalities for stage IV patients with different T/N stages**.

According to the AJCC Staging Manual tumor stage (7^th^ edition), stage IV refers to any T/any N/M1, which means that all of the patients with mUTUC were in this range. Therefore, we grouped the stage IV patients according to the previous results, and performed Kaplan–Meier curves with some new combinations. Because the sample size of patients who underwent radiotherapy in each subgroup after stratification was too small, the treatment modalities included only surgery and chemotherapy. Since fewer than two patients with each staging T1/2/NX, TX/N1/2/3, and TX/N0 underwent surgery, K–M analysis could not be performed. The treatment strategies of stage IV patients with different T/N stages are shown in Table 3. For metastatic patients with T3/N0, T3/N1/2/3, T4/N0, and T4/N1/2/3, the Kaplan–Meier curves of patients undergoing surgery and chemotherapy and chemotherapy alone are shown in Figures S12 (*p* = 0.0185), S13, S14, and S15.

**TABLE 3:** Treatment strategies of stage IV patients with different T/N stages

IV Stage	T1/2	N0/1/2/3	Surgery + chemotherapy	Figure S5/Figure S6/Figure S9
		NX	—	—
	T3	N0	Surgery + chemotherapy	Figure S12 (*p* = 0.0185)
		N1/2/3	No difference between surgery + chemotherapy and chemotherapy	Figure S13
		NX	Chemotherapy	Table S5/Table S10
	T4	N0/1/2/3	No difference between surgery + chemotherapy and chemotherapy	Figure S14/Figure S15
		NX	Chemotherapy	Table S6/Table S10
	TX	N0/1/2/3	—	—
		NX	Chemotherapy	Table S7/S10

## 4 DISCUSSION

Metastatic upper urinary tract urothelial carcinoma (mUTUC) accounts for only 10% of UTUCs, but its incidence has been increasing in the past 30 years.^3^ As mentioned in a literature review, the role of metastasis in predicting the outcome of urological cancers, such as prostate cancer,^16^ bladder cancer,^14^, ^17^ and kidney cancer,^18^ has been increasingly noted. Therefore, it is necessary to investigate the independent prognostic factors affecting metastatic UTUC and to develop models to predict the OS and select individualized treatment strategies accordingly.

Many studies have shown that distant metastasis was associated with much worse OS in UTUC and patients with distant metastasis had a higher probability of receiving chemotherapy instead of surgery at the primary site, which were revealed by our results as well.^6^, ^11^, ^12^, ^13^, ^16^, ^17^, ^18^ In terms of the distribution of distal metastasis sites, the study by Tanaka N. et al. showed that the predominant site of distant metastasis was the lung, which was more common than the liver and bone.^19^ The results of that study are consistent with ours. Moreover, our study demonstrated that as long as liver metastasis occurred, regardless of whether there were simultaneous metastases to other sites, the OS would be very poor. Dong F. et al. also observed a similar phenomenon, in which liver metastases predicted a worse OS, independent of the number of metastases, in their study of metastatic bladder cancer and metastatic UTUC.^10^, ^17^ This may be because liver metastases were more likely to cause liver failure, thus making it difficult for patients to tolerate chemotherapy‐related toxicity and other treatments. In consequence, being alert to the appearance of liver metastases through regular reviews or follow‐up and discussions of whether surgical resection of metastases at distal sites is a beneficial subject we need to investigate further.

According to the univariate and multivariate Cox regression analyses after PSM, we found that T stage, N stage, hepatic metastasis, surgery, and chemotherapy were independent prognostic factors for mUTUC. The studies of both Lughezzani G. et al and Margulis V.et al demonstrated that T stage was indeed a significant prognostic factor related to oncologic outcomes.^4^, ^20^ A higher T stage predicts a worse OS in patients with metastatic UTUC, and the patients with TX stage had the worst prognosis. In addition, Burger M. et al. confirmed that N stage was also related to OS in locally advanced UTUC.^21^ Our findings agree that for patients with distal metastasis, N stage was also related to OS. Metastatic patients with N0 stage disease have a better prognosis than N1/N2/N3 and NX patients, while NX is a sign of the worst prognosis.

For patients with metastatic UTUC (mUTUC), chemotherapy has been the optimal choice for a long time, and RNU is recommended for palliative treatment, with the aim of controlling the symptoms of the disease. However, our analysis revealed that both surgery and chemotherapy are treatment methods that can significantly improve the prognosis of patients with mUTUC. It is noteworthy that some study suggested these benefits may be limited to those with only one metastatic site, which was not confirmed by our study.^12^, ^22^ Further analysis of subgroups of T stage, N stage, and different metastatic sites revealed that the survival of patients with T1/T2, N0/N1/N2/N3, metastasis including liver, and metastasis including bone could be improved by a combination of surgery and chemotherapy, while for patients with T3/T4/TX, NX, metastasis including lung, and metastasis including distant lymph nodes, chemotherapy alone was the better choice to improve their OS. This result suggests that surgery could have an important role in the treatment strategies of patients with many types of distant metastases.^11^ It was proven that radiotherapy was useful for patients with N1/N2/N3, which is to our knowledge is the first study to confirm a role of radiotherapy in the treatment of UTUC, but more samples and studies are needed to confirm this conclusion. Stage IV refers to any T/any N/M1 according to the AJCC Staging Manual tumor stage (7^th^ edition). Therefore, a more precise treatment strategy was established for stage IV patients based on our stratified analysis of T/N stage. For patients with T1/2&N0/1/2/3 and T3&N0, their prognosis could be significantly improved by the combination of surgery and chemotherapy, and for patients with T3/4/X&NX, their prognosis could just be improved by chemotherapy alone. We hope it will provide more personalized options for patients with mUTUC.

Although this research was performed rigorously, it still has many limitations. First, we cannot know the sequence of chemotherapy and surgery because this information is not recorded in the SEER database. Some studies have shown that neoadjuvant chemotherapy before radical nephroureterectomy might provide better survival outcomes for patients with locally advanced UTUC.^23^, ^24^ Second, the SEER database lacks other important information about smoking status^25^ and a history of using aristolochic acid, which are both closely related to urothelial carcinomas.^26^ Third, our experiment did not eliminate all variable bias between the surgery and non‐surgery groups after PSM.

## 5 CONCLUSION

Our SEER database analysis was a retrospective study investigating the influence of surgery on primary site to the metastatic renal pelvis and ureter cancers. Distant metastasis is a factor suggesting a poor OS of renal pelvis and ureter cancer. For patients with mUTUC, there were five independent prognostic factors: T stage, N stage, hepatic metastasis, surgery, and chemotherapy. A nomogram was established to predict the 4, 8, and 12‐months survival of patients with distant metastasis. Although chemotherapy alone can improve the OS of patients with distant metastasis, it was less efficient than combining surgery and chemotherapy for patients with T1/T2, N0/N1/N2/N3 stage, and metastasis including the liver and bone. However, for patients with T3/T4/TX, NX, and metastasis including lung and distant lymph nodes, chemotherapy alone was still the best choice to improve the prognosis. It is worth mentioning that radiotherapy was proven to be useful for patients with N1/N2/N3 disease. Moreover, more precise treatment strategies were provided for stage IV patients with different T/N stages.

## CONFLICT OF INTEREST

The authors declare no potential conflict of interest.

## ETHICAL APPROVAL STATEMENT

All data were from the SEER database. The SEER database is publicly available and considered.

## Supporting information

Fig S1‐15

Table S1

Table S2

Table S3

Table S4

Table S5

Table S6

Table S7

Table S8

Table S9

Table S10

Table S11

Table S12

Table S13

Table S14

## Data Availability

The data from present study are available in the Surveillance, Epidemiology, and End Results, https://seer.cancer.gov.
