# Supplementary material for: The role of surgery on primary site in metastatic upper urinary tract urothelial carcinoma and a nomogram for predicting the survival of patients with metastatic upper urinary tract urothelial carcinoma
Source: Cancer Med. 2021 Oct 14;10(22):8079–90. doi: 10.1002/cam4.4327 (PMC8607251; doi:10.1002/cam4.4327)
Supplement: Supplementary file 1 — Fig S1‐15 [file CAM4-10-8079-s009.docx]

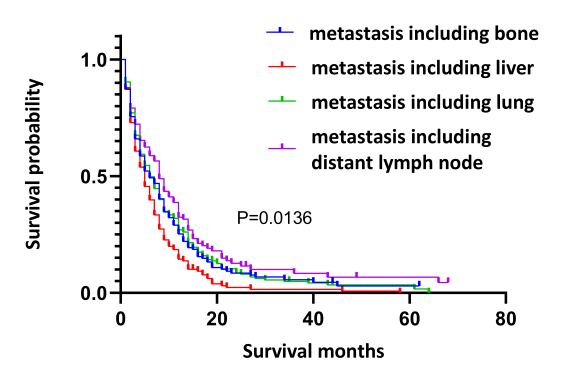

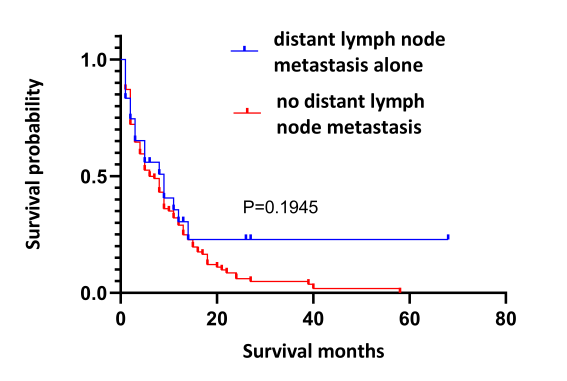

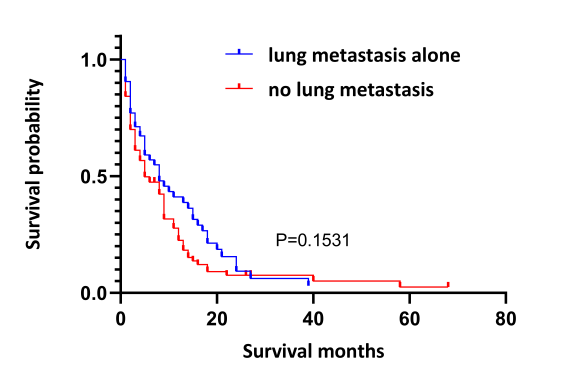

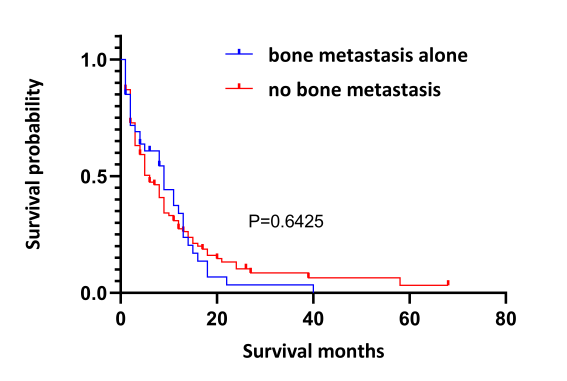

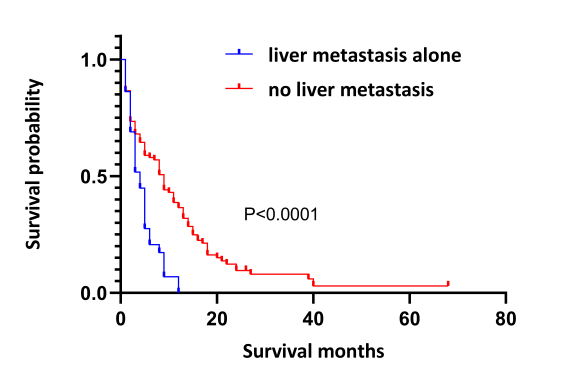

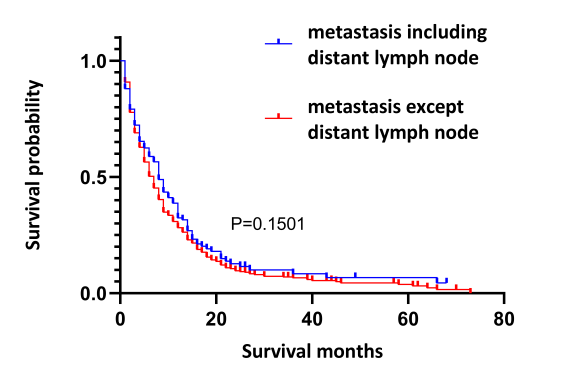

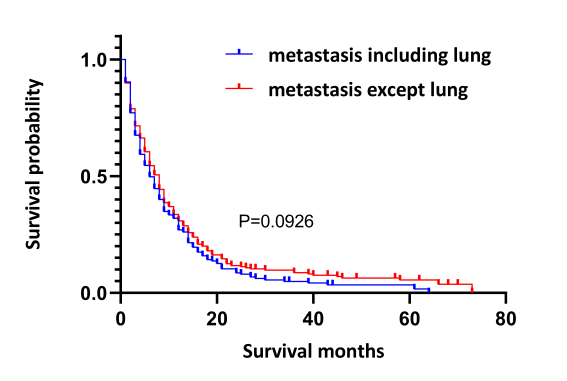

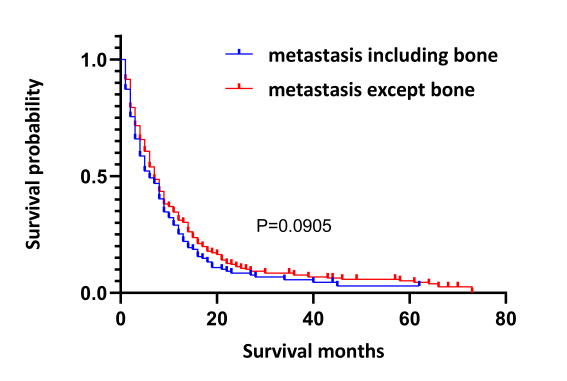


**Figure S1** a. Kaplan-Meier survival curves for metastatic upper urinary tract urothelial carcinoma with four different metastatic sites including bone, liver, lung, distant lymph node. (P=0.0136)

b/c/d. Kaplan-Meier survival curves for metastatic upper urinary tract urothelial carcinoma with metastasis including bone(b), lung(c) and distant lymph node(d).

**h**

**g**

**e**

**f**

**b**

**a**

**c**

**d**

e. Kaplan-Meier survival curves for metastatic upper urinary tract urothelial carcinoma with only one site of metastasis including liver (P<0.0001)

f/g/h. Kaplan-Meier survival curves for metastatic upper urinary tract urothelial carcinoma with only one site of metastasis including bone(f), lung(g) and distant lymph node(h).


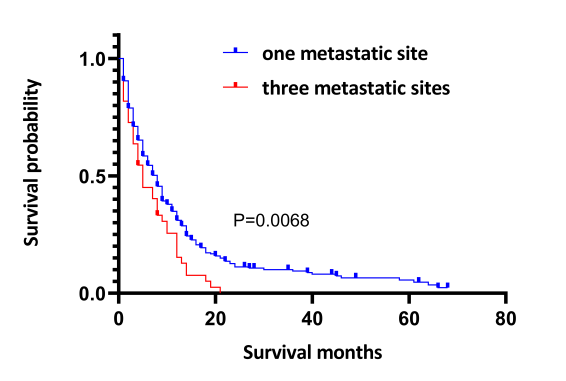

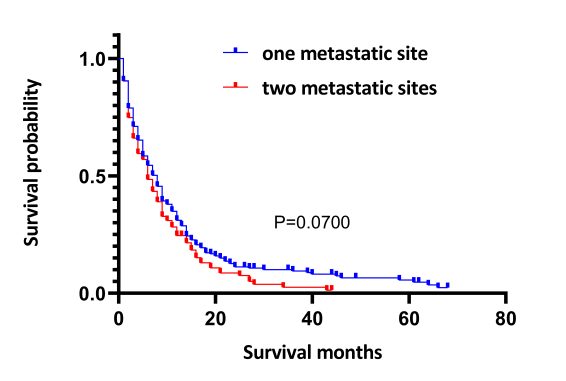


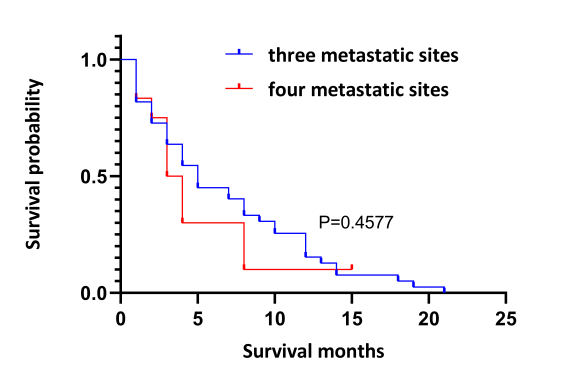

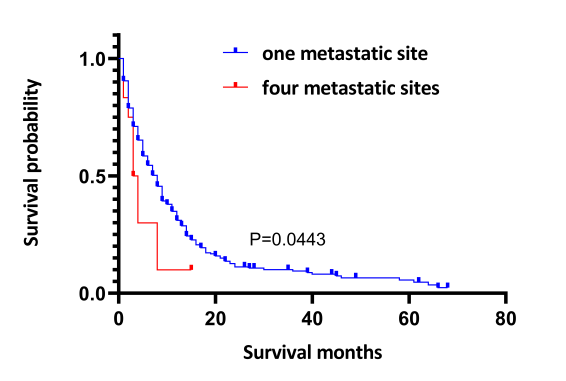


**b**

**d**

**a**

**c**


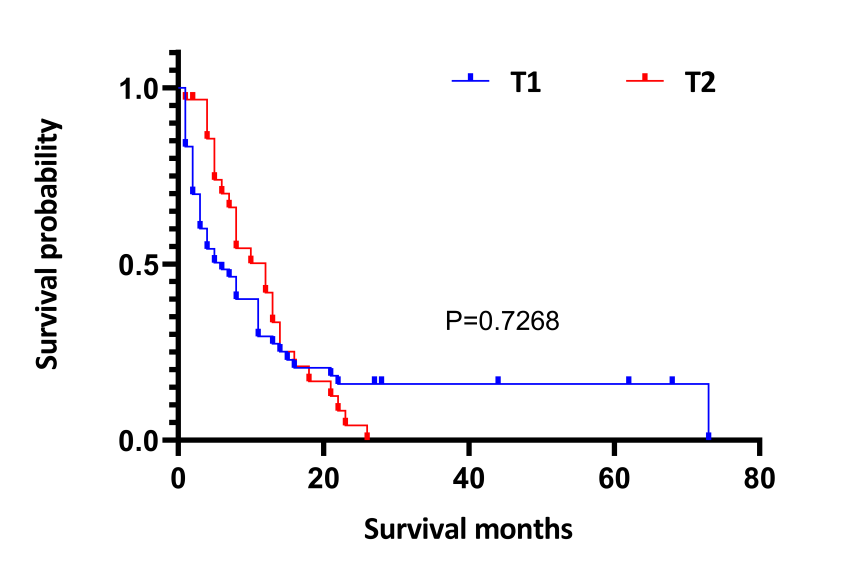


**Figure S3** Kaplan-Meier survival curves for metastatic upper urinary tract urothelial carcinoma with T1 stage and T2 stage

**Figure S2** a. Kaplan-Meier survival curves for metastatic upper urinary tract urothelial carcinoma with one site of metastasis and two sites of metastasis(a), one site of metastasis and three sites of metastasis (b, P=0.0068), one site of metastasis and four sites of metastasis (c, P=0.0443), three sites of metastasis and four sites of metastasis(d)


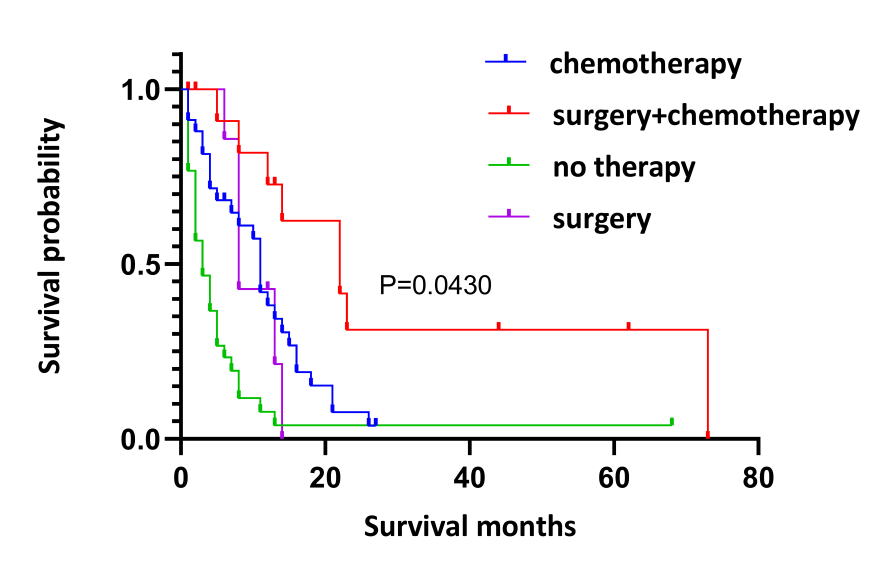

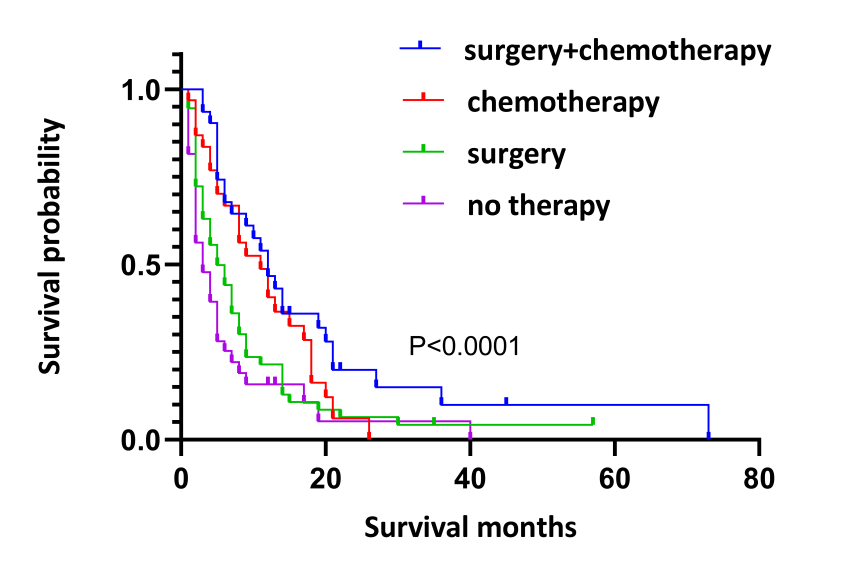


**Figure S5** Kaplan-Meier survival curves for metastatic urinary tract urothelial carcinoma with N0 stage undergoing different therapy modes including surgery and chemotherapy, surgery alone, chemotherapy alone, and no therapy (P<0.0001)

**Figure S4** Kaplan-Meier survival curves for metastatic urinary tract urothelial carcinoma with T1/2 stage undergoing different therapy modes including surgery and chemotherapy, surgery alone, chemotherapy alone, and no therapy (P= P=0.0430)


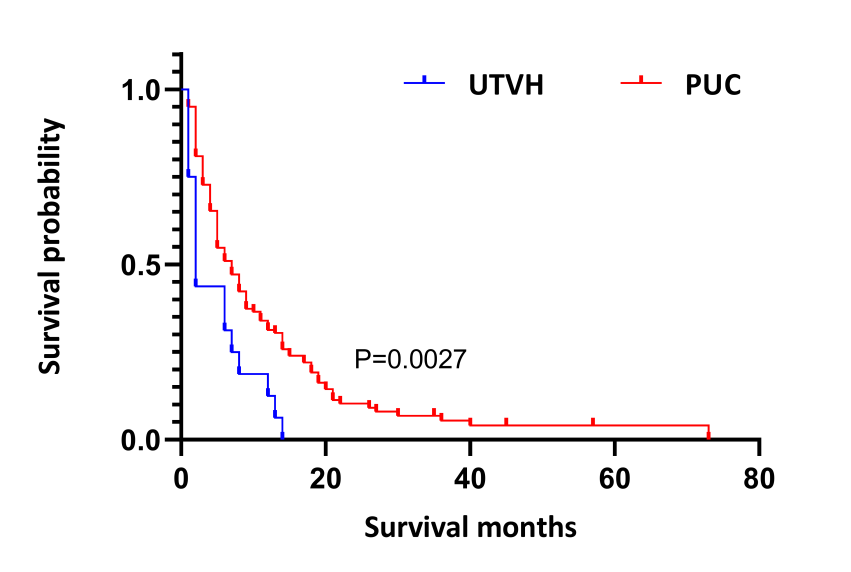

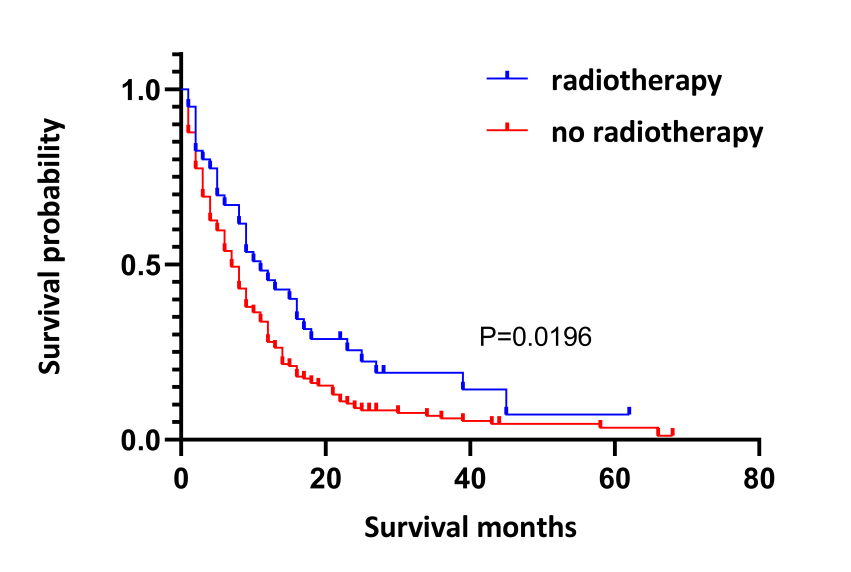


**Figure S7** Kaplan-Meier survival curves for metastatic upper urinary tract urothelial carcinoma in N1/2/3 stage with radiotherapy and without radiotherapy (P=0.0196)

**Figure S6** Kaplan-Meier survival curves for metastatic upper urinary tract urothelial carcinoma in N0 stage with PUC and UTVH (P=0.0027)

§. PUC: pure upper urinary tract urothelial cell carcinoma; UTVH: upper urinary tract tumors with variant histology


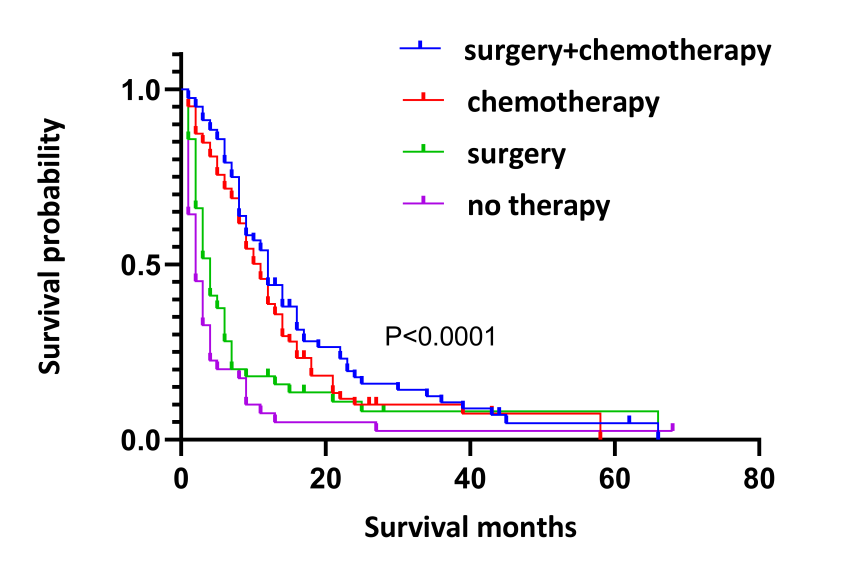


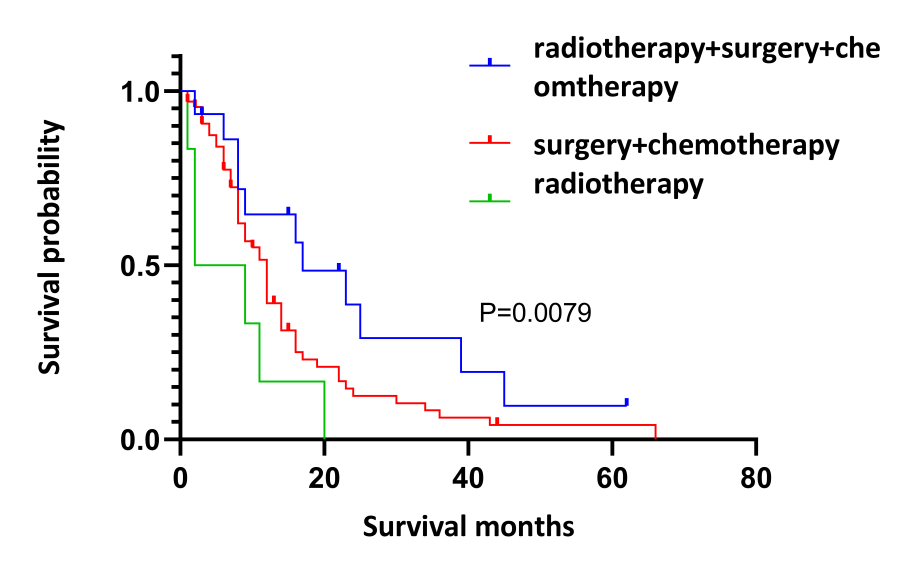


**Figure S9** Comprehensive Kaplan-Meier analyses for metastatic urinary tract urothelial carcinoma with N1/2/3 stage undergoing three therapy modes including the combination of surgery, chemotherapy and radiotherapy, surgery alone, chemotherapy alone, and radiotherapy alone (P=0.0079).

**Figure S8** Kaplan-Meier survival curves for metastatic urinary tract urothelial carcinoma with N1/2/3 stage undergoing different therapy modes including surgery and chemotherapy, surgery alone, chemotherapy alone, and no therapy (P<0.0001)


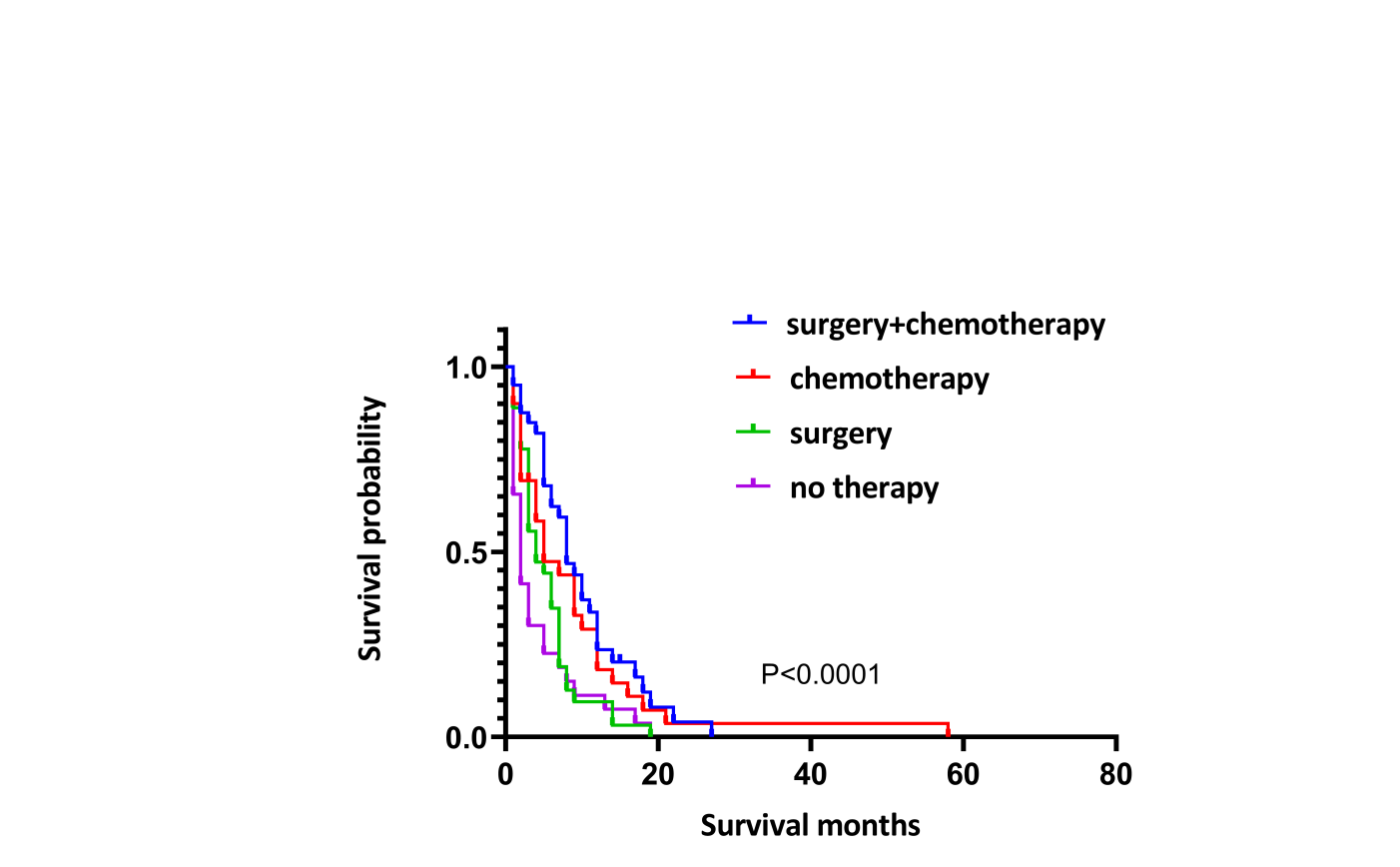


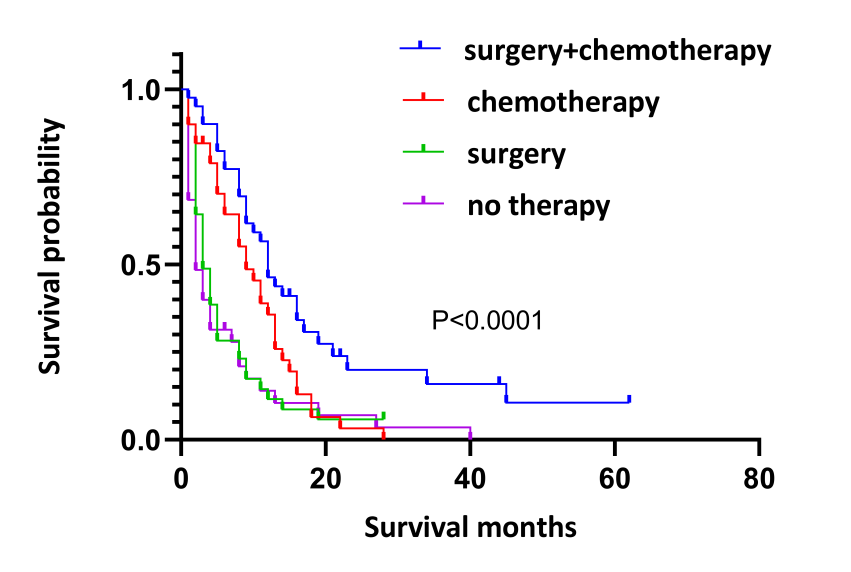


**Figure S11** Kaplan-Meier survival curves for metastatic urinary tract urothelial carcinoma with bone metastasis undergoing different therapy modes including surgery and chemotherapy, surgery alone, chemotherapy alone, and no therapy (P<0.0001)

**Figure S10** Kaplan-Meier survival curves for metastatic urinary tract urothelial carcinoma with liver metastasis undergoing different therapy modes including surgery and chemotherapy, surgery alone, chemotherapy alone, and no therapy (P<0.0001)


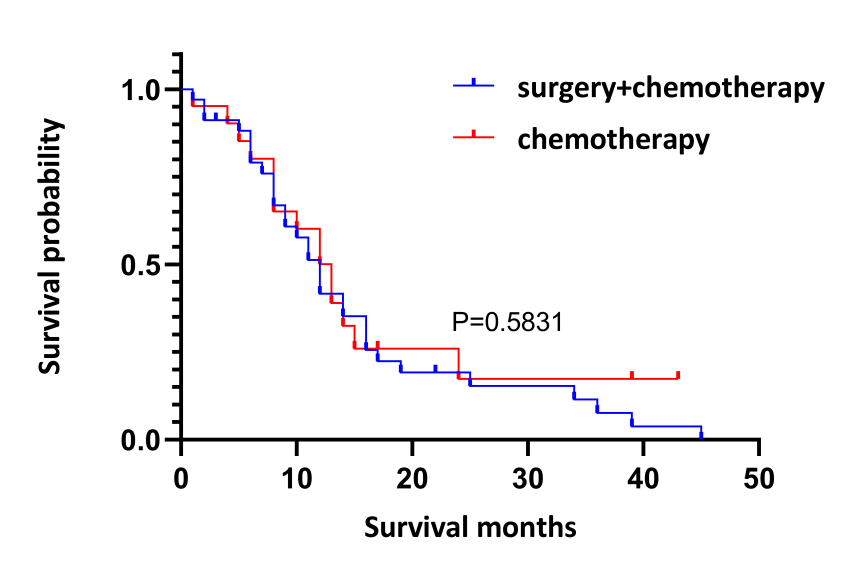

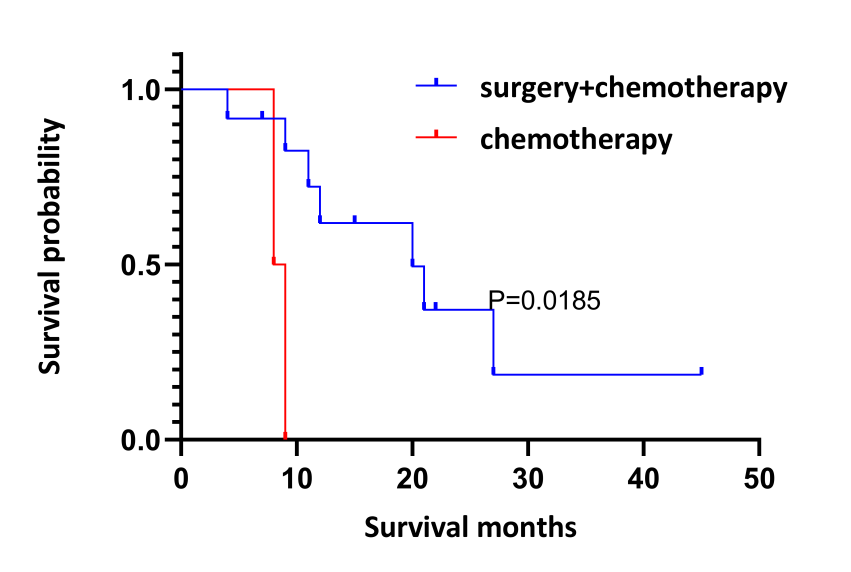


**Figure S13** Kaplan-Meier survival curves for metastatic urinary tract urothelial carcinoma with T3 and N1/2/3 stage undergoing surgery and chemotherapy and chemotherapy alone

**Figure S12** Kaplan-Meier survival curves for metastatic urinary tract urothelial carcinoma with T3 and N0 stage undergoing surgery and chemotherapy and chemotherapy alone (P=0.0185)


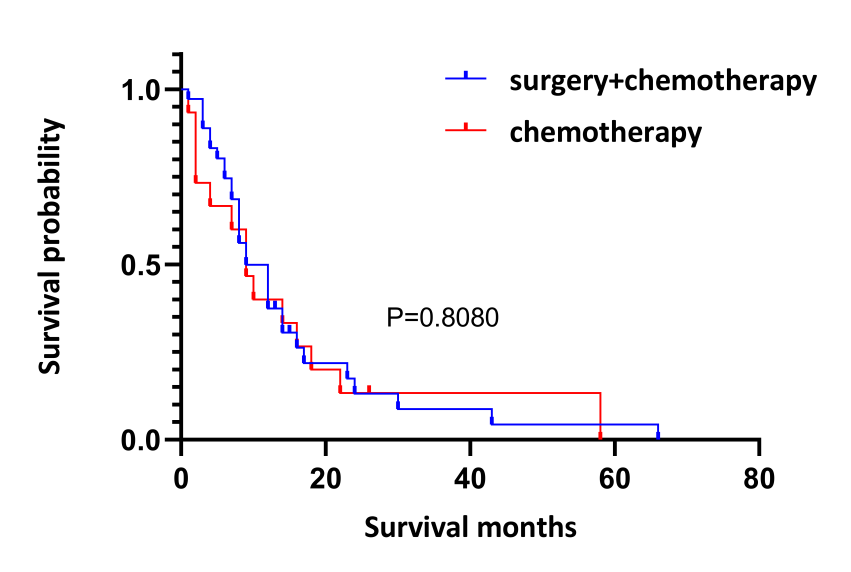

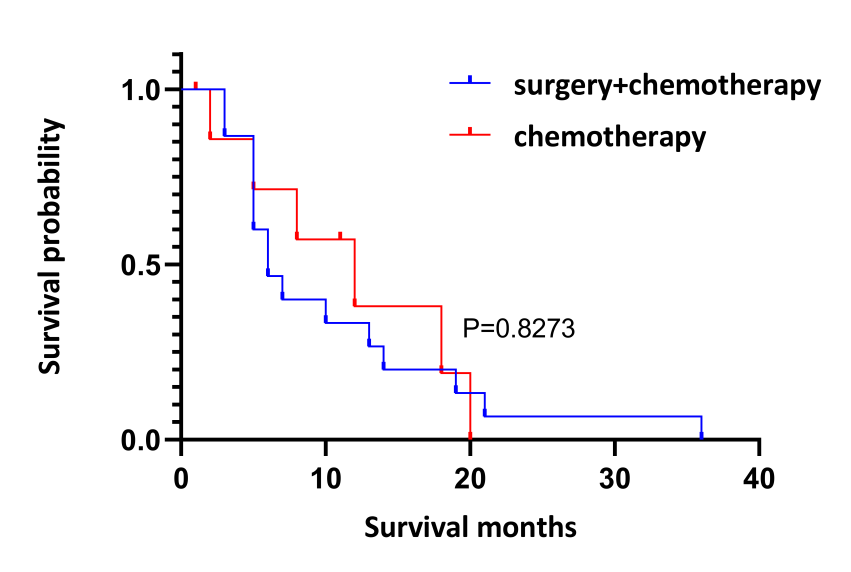


**Figure S15** Kaplan-Meier survival curves for metastatic urinary tract urothelial carcinoma with T4 and N1/2/3 stage undergoing surgery and chemotherapy and chemotherapy alone

**Figure S14** Kaplan-Meier survival curves for metastatic urinary tract urothelial carcinoma with T4 and N0 stage undergoing surgery and chemotherapy and chemotherapy alone

**e**

**a**

**c**
