# Supplementary material for: The role of surgery on primary site in metastatic upper urinary tract urothelial carcinoma and a nomogram for predicting the survival of patients with metastatic upper urinary tract urothelial carcinoma
Source: Cancer Med. 2021 Oct 14;10(22):8079–90. doi: 10.1002/cam4.4327 (PMC8607251; doi:10.1002/cam4.4327)
Supplement: Supplementary file 2 — Table S1 [file CAM4-10-8079-s011.docx]

Table S1 Clinicopathological features of upper urinary tract urothelial carcinoma patients with and without metastasis

| **Covariates** | Total (n=6724) | No-metastasis (n=6096) | Metastasis (n=628) | P value |
| --- | --- | --- | --- | --- |
| **Age at diagnosis (years)** |  |  |  | **0.062** |
| **<70** | 2447(36.4%) | 2197(36.0%) | 250(39.8%) |  |
| **>=70** | 4277(63.6%) | 3899(64.0%) | 378(60.2%) |  |
| **Race** |  |  |  | **0.269** |
| **Black** | 309(4.6%) | 275(4.5%) | 34(5.4%) |  |
| **Other** | 539(8.0%) | 481(7.9%) | 58(9.2%) |  |
| **White** | 5876(87.4%) | 5340(87.6%) | 536(85.4%) |  |
| **Histologic type** |  |  |  | **<0.0001** |
| **PUC** | 6428(95.6%) | 5858(96.1%) | 570(90.8%) |  |
| **UTVH** | 238(3.9%) | 58(9.2%) | 296(4.4%) |  |
| **Grade** |  |  |  | **<0.0001** |
| **I** | 268(4.0%) | 260(4.3%) | 8(1.3%) |  |
| **II** | 838(12.5%) | 796(13.1%) | 42(67%) |  |
| **III** | 1559(23.2%) | 1350(22.1%) | 2.9(33.3%) |  |
| **IV** | 3690(60.5%) | 369(58.8%) | 4059(60.4%) |  |
| **T stage** |  |  |  | **<0.0001** |
| **T0** | 1(0.0%) | 0(0.0%) | 1(0.0%) |  |
| **T1** | 2168(32.2%) | 2083(34.2%) | 85(13.5%) |  |
| **T2** | 1088(16.2%) | 1051(17.2%) | 37(5.9%) |  |
| **T3** | 2546(37.9%) | 2350(38.5%) | 196(31.2%) |  |
| **T4** | 670(10.0%) | 473(7.8%) | 197(31.4%) |  |
| **TX** | 251(3.7%) | 138(2.3%) | 113(18.0%) |  |
| **N stage** |  |  |  | **<0.0001** |
| **N0** | 5529(82.2%) | 5307(87.1%) | 222(35.4%) |  |
| **N1** | 499(7.4%) | 343(5.6%) | 156(24.8%) |  |
| **N2** | 523(7.8%) | 347(5.7%) | 176(28.0%) |  |
| **N3** | 32(0.5%) | 20(0.3%) | 12(1.9%) |  |
| **NX** | 141(2.1%) | 79(13%) | 62(9.9%) |  |
| **Surgery** |  |  |  | **<0.0001** |
| **No** | 1085(16.1%) | 776(12.7%) | 309(49.2%) |  |
| **Yes** | 5639(83.9%) | 5320(87.3%) | 319(50.8%) |  |
| **Surgery about regional lymph nodes** |  |  |  | **0.004** |
| **No surgery** | 4768(70.9%) | 4317(70.8%) | 451(71.8%) |  |
| **Only biopsy** | 46(0.7%) | 34(0.6%) | 12(1.9%) |  |
| **Surgery and lymph node removed** | 1907(28.4%) | 1742(28.6%) | 165(26.3%) |  |
| **Radiotherapy** |  |  |  | **<0.0001** |
| **Refused/None/unknown** | 6363(94.6%) | 5831(95.7%) | 532(84.7%) |  |
| **Yes** | 361(.4%) | 265(4.3%) | 96(15.3%) |  |
| **Chemotherapy** |  |  |  | **<0.0001** |
| **No/unknown** | 5116(76.1%) | 4821(79.1%) | 295(47.0%) |  |
| **Yes** | 1608(23.9%) | 1275(20.9%) | 333(53.0%) |  |

§. PUC: pure upper urinary tract urothelial cell carcinoma; UTVH: upper urinary tract tumors with variant histology
