# Supplementary material for: The role of surgery on primary site in metastatic upper urinary tract urothelial carcinoma and a nomogram for predicting the survival of patients with metastatic upper urinary tract urothelial carcinoma
Source: Cancer Med. 2021 Oct 14;10(22):8079–90. doi: 10.1002/cam4.4327 (PMC8607251; doi:10.1002/cam4.4327)
Supplement: Supplementary file 3 — Table S2 [file CAM4-10-8079-s012.docx]

Table S2 Univariable and multivariable Cox regression modle analyses for overall survival of all patients with upper urinary tract urothelial carcinoma

| variables | level | univariable | | | multivariable | | |
| --- | --- | --- | --- | --- | --- | --- | --- |
|  |  | P value | HR | 95%CI | P value | HR | 95%CI |
| **Age at diagnosis (years)** | 70-79 | <0.0001 |  |  | <0.0001 |  |  |
|  | >79 | <0.0001 | 1.743 | 1.610-1.886 | <0.0001 | 1.829 | 1.690-1.980 |
| **Race** | Black(ref) | 0.414 |  |  |  |  |  |
|  | White | 0.509 | 0.933 | 0.759-1.147 |  |  |  |
|  | Other | 0.215 | 0.899 | 0.759-1.064 |  |  |  |
| **Histologic type** | PUC(ref) | <0.0001 |  |  |  |  |  |
|  | UTVH | <0.0001 | 1.862 | 1.602-2.165 |  |  |  |
| **Grade** | I (ref) | <0.0001 |  |  |  |  |  |
|  | II | 0.798 | 1.032 | 0.812-1.311 |  |  |  |
|  | III | <0.0001 | 2.178 | 1.751-2.709 |  |  |  |
|  | IV | <0.0001 | 1.817 | 1.468-2.248 |  |  |  |
| **T stage** | T0 (ref) | <0.0001 |  |  |  |  |  |
|  | T1 | 0.783 | 6.449 | 5.968-7.242 |  |  |  |
|  | T2 | 0.761 | 2.932 | 2.538-3.127 |  |  |  |
|  | T3 | 0.733 | 3.020 | 2.887-3.454 |  |  |  |
|  | T4 | 0.659 | 8.034 | 6.793-9.567 |  |  |  |
|  | TX | 0.675 | 7.946 | 6.754-8.222 |  |  |  |
| **N stage** | N0(ref) | <0.0001 |  |  |  |  |  |
|  | N1 | <0.0001 | 2.831 | 2.528-3.170 |  |  |  |
|  | N2 | <0.0001 | 2.989 | 2.669-3.347 |  |  |  |
|  | N3 | <0.0001 | 4.257 | 2.869-6.317 |  |  |  |
|  | NX | <0.0001 | 3.111 | 2.548-3.798 |  |  |  |
| **M stage** | No(ref) | <0.0001 |  |  | <0.0001 |  |  |
|  | Yes | <0.0001 | 5.449 | 4.944-6.005 | <0.0001 | 5.723 | 5.191-6.309 |
| **Radiotherapy** | No/unknown | <0.0001 |  |  |  |  |  |
|  | Yes | <0.0001 | 2.057 | 1.806-2.342 |  |  |  |
| **Chemotherapy** | No (ref) | <0.0001 |  |  | <0.0001 |  |  |
|  | Yes | <0.0001 | 0.372 | 0.266-0.488 | <0.0001 | 0.421 | 0.353-0.502 |
| **Surgery** | No (ref) | <0.0001 |  |  | <0.0001 |  |  |
|  | Yes | <0.0001 | 0.699 | 0.589-0.831 | <0.0001 | 0.683 | 0.551-0.846 |

§. PUC: pure upper urinary tract urothelial cell carcinoma; UTVH: upper urinary tract tumors with variant histology
