# Supplementary material for: The role of surgery on primary site in metastatic upper urinary tract urothelial carcinoma and a nomogram for predicting the survival of patients with metastatic upper urinary tract urothelial carcinoma
Source: Cancer Med. 2021 Oct 14;10(22):8079–90. doi: 10.1002/cam4.4327 (PMC8607251; doi:10.1002/cam4.4327)
Supplement: Supplementary file 4 — Table S3 [file CAM4-10-8079-s003.docx]

Table S3 Univariable and multivariable Cox regression model analyses for overall survival of 628 patients with metastatic upper urinary tract urothelial carcinoma before PSM

| variables | level | univariable | | | multivariable | | |
| --- | --- | --- | --- | --- | --- | --- | --- |
|  |  | P value | HR | 95%CI | P value | HR | 95%CI |
| **Age at diagnosis (years)** | 70-79 | 0.001 |  |  |  |  |  |
|  | >79 | 0.001 | 1.362 | 1.142-1.625 |  |  |  |
| **Race** | Black(ref) | 0.969 |  |  |  |  |  |
|  | White | 0.866 | 0.959 | 0.589-1.561 |  |  |  |
|  | Other | 0.981 | 0.995 | 0.660-1.501 |  |  |  |
| **Histologic type** | PUC(ref) | 0.633 |  |  |  |  |  |
|  | UTVH | 0.633 | 1.072 | 0.805-1.428 |  |  |  |
| **Grade** | I (ref) | 0.177 |  |  |  |  |  |
|  | II | 0.199 | 1.855 | 0.722-4.765 |  |  |  |
|  | III | 0.070 | 2.280 | 0.936-2.280 |  |  |  |
|  | IV | 0.120 | 2.022 | 0.833-4.906 |  |  |  |
| **T stage** | T1 (ref) | **<0.0001** |  |  | 0.007 |  |  |
|  | T2 | 0.853 | 0.960 | 0.626-1.473 | 0.495 | 1.162 | 0.755-1.788 |
|  | T3 | 0.433 | .891 | 0.668-1.189 | 0.500 | 1.114 | 0.814-1.525 |
|  | T4 | 0.081 | 1.289 | 0.970-1.714 | 0.010 | 1.483 | 1.100-2.000 |
|  | TX | 0.002 | 1.649 | 1.202-2.263 | 0.007 | 1.556 | 1.131-2.141 |
| **N stage** | N0(ref) | 0.069 |  |  |  |  |  |
|  | N1/N2/N3 | 0.062 | 0.752 | 0.558-1.014 |  |  |  |
|  | NX | 0.021 | 0.713 | 535-0.950 |  |  |  |
| **Radiotherapy** | No/unknown | 0.174 |  |  |  |  |  |
|  | Yes | 0.174 | 0.848 | 0.668-1.076 |  |  |  |
| **Chemotherapy** | No (ref) | <0.0001 |  |  | <0.0001 |  |  |
|  | Yes | <0.0001 | 0.437 | 0.367-0.521 | <0.0001 | 0.429 | 0.360-0.512 |
| **Surgery** | No (ref) | <0.0001 |  |  | 0.001 |  |  |
|  | Yes | <0.0001 | 0.683 | 0.575-0.811 | 0.001 | 0.697 | 0.562-0.866 |
| **Surgery about regional lymph nodes** | No surgery (ref) | 0.012 |  |  |  |  |  |
|  | Only biopsy | 0.516 | 0.803 | 0.415-1.556 |  |  |  |
|  | Surgery and lymph node removed | 0.003 | 0.742 | 0.608-0.904 |  |  |  |
| **Metastatic including bone** | No(ref) | 0.083 |  |  |  |  |  |
|  | Yes | 0.083 | 1.175 | 0.9791.411 |  |  |  |
| **Metastatic including brain** | No(ref) | 0.129 |  |  |  |  |  |
|  | Yes | 0.129 | 1.560 | 0.879-2.770 |  |  |  |
| **Metastatic including liver** | No(ref) | <0.0001 |  |  | <0.0001 |  |  |
|  | Yes | <0.0001 | 1.800 | 1.497-2.165 | <0.0001 | 1.619 | 1.340-1.957 |
