# Supplementary material for: The role of surgery on primary site in metastatic upper urinary tract urothelial carcinoma and a nomogram for predicting the survival of patients with metastatic upper urinary tract urothelial carcinoma
Source: Cancer Med. 2021 Oct 14;10(22):8079–90. doi: 10.1002/cam4.4327 (PMC8607251; doi:10.1002/cam4.4327)
Supplement: Supplementary file 5 — Table S4 [file CAM4-10-8079-s015.docx]

Table S4 Univariable and multivariable Cox regression model analyses for overall survival of metastatic upper urinary tract urothelial carcinoma with T1/2 stage after PSM

| variables | level | univariable | | | multivariable | | |
| --- | --- | --- | --- | --- | --- | --- | --- |
|  |  | P value | HR | 95%CI | P value | HR | 95%CI |
| **Age at diagnosis (years)** | 70-79 | 0.185 |  |  |  |  |  |
|  | >79 | 0.185 | 1.419 | 0.846-2.381 |  |  |  |
| **Race** | Black(ref) | 0.740 |  |  |  |  |  |
|  | White | 0.443 | 0.577 | 0.141-2.352 |  |  |  |
|  | Other | 0.495 | 0.664 | 0.205-2.151 |  |  |  |
| **Histologic type** | PUC(ref) | 0.461 |  |  |  |  |  |
|  | UTVH | 0.461 | 0.790 | 0.421-1.480 |  |  |  |
| **N stage** | N0(ref) | 0.010 |  |  |  |  |  |
|  | N1/N2/N3 | 0.123 | 0.464 | 0.175-1.231 |  |  |  |
|  | NX | 0.008 | 0.261 | 0.096-0.708 |  |  |  |
| **Radiotherapy** | No/unknown | 0.383 |  |  |  |  |  |
|  | Yes | 0.383 | 0.757 | 0.405-1.415 |  |  |  |
| **Chemotherapy** | No (ref) | 0.002 |  |  | 0.002 |  |  |
|  | Yes | 0.002 | 0.467 | 0.288-0.755 | 0.002 | 0.457 | 0.278-0.752 |
| **Surgery** | No (ref) | <0.0001 |  |  | <0.0001 |  |  |
|  | Yes | <0.0001 | 0.317 | 0.167-0.600 | <0.0001 | 0.401 | 0.179-0.646 |
| **Surgery about regional lymph nodes** | No surgery (ref) | 0.071 |  |  |  |  |  |
|  | Only biopsy | 0.880 | 0.896 | 0.216-3.710 |  |  |  |
|  | Surgery and lymph node removed | 0.022 | 0.191 | 0.046-0.784 |  |  |  |
| **Metastatic including bone** | No(ref) | 0.461 |  |  |  |  |  |
|  | Yes | 0.461 | 0.828 | 0.501-1.368 |  |  |  |
| **Metastatic including liver** | No(ref) | 0.005 |  |  |  |  |  |
|  | Yes | 0.005 | .204 | 1.276-3.806 | 0.015 |  |  |
| **Metastatic including lung** | No(ref) | 0.063 |  |  | 0.015 | 2.065 | 1.148-3.713 |
|  | Yes | 0.063 | 1.593 | 0.975-1.603 |  |  |  |
| **Metastatic including distant lymph node** | No(ref) | 0.645 |  |  |  |  |  |
|  | Yes | 0.645 | 0.885 | 0.525-1.490 |  |  |  |
| **The number of metastatic sites** | One or two sites (ref) | 0.066 |  |  |  |  |  |
|  | Three or four sites | 0.158 | 1.942 | 0.774-4.877 |  |  |  |
|  | Distant metastatic sites can’t be assessed | 0.021 | 4.162 | 1.240-13.9710 |  |  |  |

§. PUC: pure upper urinary tract urothelial cell carcinoma; UTVH: upper urinary tract tumors with variant histology
