# Supplementary material for: The role of surgery on primary site in metastatic upper urinary tract urothelial carcinoma and a nomogram for predicting the survival of patients with metastatic upper urinary tract urothelial carcinoma
Source: Cancer Med. 2021 Oct 14;10(22):8079–90. doi: 10.1002/cam4.4327 (PMC8607251; doi:10.1002/cam4.4327)
Supplement: Supplementary file 6 — Table S5 [file CAM4-10-8079-s007.zip › cam44327-sup-0006-TableS5/cam44327-sup-0006-TableS5-1.docx]

Table S5 Univariable and multivariable Cox regression model analyses for overall survival of metastatic upper urinary tract urothelial carcinoma with T3 stage after PSM

| variables | level | univariable | | | multivariable | | |
| --- | --- | --- | --- | --- | --- | --- | --- |
|  |  | P value | HR | 95%CI | P value | HR | 95%CI |
| **Age at diagnosis (years)** | 70-79 | 0.721 |  |  |  |  |  |
|  | >79 | 0.721 | 0.936 | 0.652-1.344 |  |  |  |
| **Race** | Black(ref) | 0.648 |  |  |  |  |  |
|  | White | 0.671 | 0.813 | 0.314-2.109 |  |  |  |
|  | Other | 0.387 | 0.713 | 0.331-1.536 |  |  |  |
| **Histologic type** | PUC(ref) | 0.277 |  |  |  |  |  |
|  | UTVH | 0.277 | 1.393 | 0.766-2.532 |  |  |  |
| **N stage** | N0(ref) | 0.632 |  |  |  |  |  |
|  | N1/N2/N3 | 0.434 | 0.770 | 0.401-1.481 |  |  |  |
|  | NX | 0.761 | 0.909 | 0.493-1.677 |  |  |  |
| **Radiotherapy** | No/unknown | 0.122 |  |  |  |  |  |
|  | Yes | 0.122 | 0.689 | 0.430-1.105 |  |  |  |
| **Chemotherapy** | No (ref) | <0.0001 |  |  | <0.0001 |  |  |
|  | Yes | <0.0001 | 0.453 | 0.316-0.650 | <0.0001 | 0.477 | 0.332-0.687 |
| **Surgery** | No (ref) | 0.505 |  |  |  |  |  |
|  | Yes | 0.505 | 0.861 | 0.554-1.337 |  |  |  |
| **Surgery about regional lymph nodes** | No surgery (ref) | 0.662 |  |  |  |  |  |
|  | Only biopsy | 0.630 | 1.414 | 0.345-5.799 |  |  |  |
|  | Surgery and lymph node removed | 0.417 | 1.166 | 0.805-1.690 |  |  |  |
| **Metastatic including bone** | No(ref) | 0.962 |  |  |  |  |  |
|  | Yes | 0.962 | 1.009 | 0.686-1.486 |  |  |  |
| **Metastatic including liver** | No(ref) | 0.002 |  |  |  |  |  |
