# Supplementary material for: The role of surgery on primary site in metastatic upper urinary tract urothelial carcinoma and a nomogram for predicting the survival of patients with metastatic upper urinary tract urothelial carcinoma
Source: Cancer Med. 2021 Oct 14;10(22):8079–90. doi: 10.1002/cam4.4327 (PMC8607251; doi:10.1002/cam4.4327)
Supplement: Supplementary file 6 — Table S5 [file CAM4-10-8079-s007.zip › cam44327-sup-0006-TableS5/cam44327-sup-0007-TableS5-2.docx]

|  | Yes | 0.002 | 1.873 | 1.266-2.771 | 0.008 |  |  |
| --- | --- | --- | --- | --- | --- | --- | --- |
| **Metastatic including lung** | No(ref) | 0.439 |  |  | 0.008 | .712 | 1.153-2.541 |
|  | Yes | 0.439 | 1.153 | 0.804-1.652 |  |  |  |
| **Metastatic including distant lymph node** | No(ref) | 0.733 |  |  |  |  |  |
|  | Yes | 0.733 | 0.931 | 0.618-1.403 |  |  |  |
| **The number of metastatic sites** | One or two sites (ref) | 0.103 |  |  |  |  |  |
|  | Three or four sites | 0.134 | 1.564 | 0.871-2.809 |  |  |  |
|  | Distant metastatic sites can’t be assessed | 0.034 | 2.498 | 1.073-5.815 |  |  |  |

§. PUC: pure upper urinary tract urothelial cell carcinoma; UTVH: upper urinary tract tumors with variant histology
