# Supplementary material for: The role of surgery on primary site in metastatic upper urinary tract urothelial carcinoma and a nomogram for predicting the survival of patients with metastatic upper urinary tract urothelial carcinoma
Source: Cancer Med. 2021 Oct 14;10(22):8079–90. doi: 10.1002/cam4.4327 (PMC8607251; doi:10.1002/cam4.4327)
Supplement: Supplementary file 7 — Table S6 [file CAM4-10-8079-s001.docx]

Table S6 Univariable and multivariable Cox regression model analyses for overall survival of metastatic upper urinary tract urothelial carcinoma with T4 stage after PSM

| variables | level | univariable | | | multivariable | | |
| --- | --- | --- | --- | --- | --- | --- | --- |
|  |  | P value | HR | 95%CI | P value | HR | 95%CI |
| **Age at diagnosis (years)** | 70-79 | 0.161 |  |  |  |  |  |
|  | >79 | 0.161 | 1.273 | 0.909-1.784 |  |  |  |
| **Race** | Black(ref) | 0.727 |  |  |  |  |  |
|  | White | 0.503 | 1.421 | 0.509-3.972 |  |  |  |
|  | Other | 0.730 | 1.174 | 0.473-2.916 |  |  |  |
| **Histologic type** | PUC(ref) | 0.284 |  |  |  |  |  |
|  | UTVH | 0.284 | 1.330 | 0.789-2.243 |  |  |  |
| **Grade** | I (ref) | 0.844 |  |  |  |  |  |
|  | II | 0.773 | 0.793 | 0.164-3.825 |  |  |  |
|  | III | 0.883 | 1.112 | 0.270-4.588 |  |  |  |
|  | IV | 0.866 | 1.128 | 0.277-4.597 |  |  |  |
| **N stage** | N0(ref) | 0.513 |  |  |  |  |  |
|  | N1/N2/N3 | 0.937 | 1.024 | 0.573-1.829 |  |  |  |
|  | NX | 0.531 | 0.837 | 0.480-1.460 |  |  |  |
| **Radiotherapy** | No/unknown | 0.703 |  |  |  |  |  |
|  | Yes | 0.703 | 0.906 | 0.545-1.505 |  |  |  |
| **Chemotherapy** | No (ref) | <00001 |  |  | <00001 |  |  |
|  | Yes | <0.0001 | 0.412 | 0.294-0.577 | <0.0001 | 0.412 | 0.294-0.577 |
| **Surgery** | No (ref) | 0.756 |  |  |  |  |  |
|  | Yes | 0.756 | 1.058 | 0.741-1.511 |  |  |  |
| **Surgery about regional lymph nodes** | No surgery (ref) | 0.299 |  |  |  |  |  |
|  | Only biopsy | 0.779 | 0.866 | 0.317-2.365 |  |  |  |
|  | Surgery and lymph node removed | 0.121 | 0.760 | 0.537-1.075 |  |  |  |
| **Metastatic including bone** | No(ref) | 0.229 |  |  |  |  |  |
|  | Yes | 0.229 | 1.237 | 0.875-1.750 |  |  |  |
| **Metastatic including liver** | No(ref) | 0.055 |  |  |  |  |  |
|  | Yes | 0.055 | 1.396 | 0.993-1.963 |  |  |  |
| **Metastatic including lung** | No(ref) | 0.575 |  |  |  |  |  |
|  | Yes | 0.575 | 1.099 | 0.790-1.529 |  |  |  |
| **Metastatic including distant lymph node** | No(ref) | 0.035 |  |  |  |  |  |
|  | Yes | 0.035 | 0.648 | 0.433-0.970 |  |  |  |
| **The number of metastatic sites** | One or two sites (ref) | 0.066 |  |  |  |  |  |
|  | Three or four sites | 0.158 | 1.942 | 0.774-4.877 |  |  |  |
|  | Distant metastatic sites can’t be assessed | 0.021 | 4.162 | 1.240-13.9710 |  |  |  |

§. PUC: pure upper urinary tract urothelial cell carcinoma; UTVH: upper urinary tract tumors with variant histology
