# Supplementary material for: The role of surgery on primary site in metastatic upper urinary tract urothelial carcinoma and a nomogram for predicting the survival of patients with metastatic upper urinary tract urothelial carcinoma
Source: Cancer Med. 2021 Oct 14;10(22):8079–90. doi: 10.1002/cam4.4327 (PMC8607251; doi:10.1002/cam4.4327)
Supplement: Supplementary file 8 — Table S7 [file CAM4-10-8079-s014.zip › cam44327-sup-0008-TableS7/cam44327-sup-0009-TableS7-1.docx]

Table S7 Univariable and multivariable Cox regression model analyses for overall survival of metastatic upper urinary tract urothelial carcinoma with TX stage after PSM

| variables | level | univariable | | | multivariable | | |
| --- | --- | --- | --- | --- | --- | --- | --- |
|  |  | P value | HR | 95%CI | P value | HR | 95%CI |
| **Age at diagnosis (years)** | 70-79 | 0.093 |  |  |  |  |  |
|  | >79 | 0.093 | 1.455 | 0.940-2.252 |  |  |  |
| **Race** | Black(ref) | 0.659 |  |  |  |  |  |
|  | White | 0.504 | 0.653 | 0.187-2.281 |  |  |  |
|  | Other | 0.362 | 0.623 | 0.226-1.722 |  |  |  |
| **Histologic type** | PUC(ref) | 0.475 |  |  |  |  |  |
|  | UTVH | 0.475 | 0.753 | 0.346-1.640 |  |  |  |
| **Grade** | I (ref) | 0.448 |  |  |  |  |  |
|  | II | 0.730 | 1.313 | 0.279-6.172 |  |  |  |
|  | III | 0.833 | 1.167 | 0.278-4.896 |  |  |  |
|  | IV | 0.476 | 1.684 | 0.402-7.045 |  |  |  |
| **N stage** | N0(ref) | 0.106 |  |  |  |  |  |
|  | N1/N2/N3 | 0.045 | 0.526 | 0.281-0.985 |  |  |  |
|  | NX | 0.070 | 0.595 | 0.340-1.043 |  |  |  |
| **Radiotherapy** | No/unknown | 0.603 |  |  |  |  |  |
|  | Yes | 0.603 | 0.854 | 0.472-1.546 |  |  |  |
| **Chemotherapy** | No (ref) | <0.0001 |  |  | <0.0001 |  |  |
|  | Yes | <0.0001 | 0.382 | 0.244-0.598 | <0.0001 | 0.377 | 0.241-0.592 |
| **Surgery** | No (ref) | 0.463 |  |  |  |  |  |
|  | Yes | 0.463 | 2.872 | 1.600-5.703 |  |  |  |
| **Surgery about regional lymph nodes** | No surgery (ref) | 0.851 |  |  |  |  |  |
|  | Only biopsy | 0.571 | 0.078 | 4.084 |  |  |  |
