# Supplementary material for: The role of surgery on primary site in metastatic upper urinary tract urothelial carcinoma and a nomogram for predicting the survival of patients with metastatic upper urinary tract urothelial carcinoma
Source: Cancer Med. 2021 Oct 14;10(22):8079–90. doi: 10.1002/cam4.4327 (PMC8607251; doi:10.1002/cam4.4327)
Supplement: Supplementary file 8 — Table S7 [file CAM4-10-8079-s014.zip › cam44327-sup-0008-TableS7/cam44327-sup-0010-TableS7-2.docx]

|  | Surgery and lymph node removed | 0.970 | 0.000 | 1.690E+271 |  |  |  |
| --- | --- | --- | --- | --- | --- | --- | --- |
| **Metastatic including bone** | No(ref) | 0.514 |  |  |  |  |  |
|  | Yes | 0.514 | 1.157 | 746-1.793 |  |  |  |
| **Metastatic including liver** | No(ref) | 0.020 |  |  |  |  |  |
|  | Yes | 0.020 | 1.746 | 1.092-2.791 | 0.017 |  |  |
| **Metastatic including lung** | No(ref) | 0.536 |  |  | 0.017 | 1.781 | 1.110-2.858 |
|  | Yes | 0.536 | 0.872 | 0.564-1.348 |  |  |  |
| **Metastatic including distant lymph node** | No(ref) | 0.635 |  |  |  |  |  |
|  | Yes | 0.635 | 1.128 | 0.686-1.855 |  |  |  |
| **The number of metastatic sites** | One or two sites (ref) | 0.363 |  |  |  |  |  |
|  | Three or four sites | 0.811 | 1.10 | 0.503-2.405 |  |  |  |
|  | Distant metastatic sites can’t be assessed | 0.255 | 1.761 | 0.665-4.664 |  |  |  |

§. PUC: pure upper urinary tract urothelial cell carcinoma; UTVH: upper urinary tract tumors with variant histology
