# Supplementary material for: The role of surgery on primary site in metastatic upper urinary tract urothelial carcinoma and a nomogram for predicting the survival of patients with metastatic upper urinary tract urothelial carcinoma
Source: Cancer Med. 2021 Oct 14;10(22):8079–90. doi: 10.1002/cam4.4327 (PMC8607251; doi:10.1002/cam4.4327)
Supplement: Supplementary file 9 — Table S8 [file CAM4-10-8079-s008.docx]

Table S8 Univariable and multivariable Cox regression model analyses for overall survival of metastatic upper urinary tract urothelial carcinoma with N0 stage after PSM

| variables | level | univariable | | | multivariable | | |
| --- | --- | --- | --- | --- | --- | --- | --- |
|  |  | P value | HR | 95%CI | P value | HR | 95%CI |
| **Age at diagnosis (years)** | 70-79 | 0.509 |  |  |  |  |  |
|  | >79 | 0.509 | 0.887 | 0.621-1.266 |  |  |  |
| **Race** | Black(ref) | 0.599 |  |  |  |  |  |
|  | White | 0.423 | 0.687 | 0.275-1.719 |  |  |  |
|  | Other | 0.311 | 0.671 | 0.309-1.454 |  |  |  |
| **Histologic type** | PUC(ref) | 0.016 |  |  | 0.028 |  |  |
|  | UTVH | 0.016 | 1.911 | 1.127-3.240 | 0.028 | 1.813 | 1.068-3.079 |
| **T stage** | T1 (ref) | 0.143 |  |  |  |  |  |
|  | T2 | 0.848 | 0.931 | 0.451-1.923 |  |  |  |
|  | T3 | 0.103 | 0.618 | 1.346-1.102 |  |  |  |
|  | T4 | 0.854 | 1.054 | 0.605-1.834 |  |  |  |
|  | TX | 0.963 | 1.015 | 0.546-1.886 |  |  |  |
| **Radiotherapy** | No/unknown | 0.629 |  |  |  |  |  |
|  | Yes | 0.629 | 1.127 | 0.694-1.831 |  |  |  |
| **Chemotherapy** | No (ref) | <0.0001 |  |  | <0.0001 |  |  |
|  | Yes | <0.0001 | 0.492 | 0.347-0.698 | <0.0001 | 0.476 | 0.335-0.677 |
| **Surgery** | No (ref) | 0.024 |  |  | 0.031 |  |  |
|  | Yes | 0.024 | 0.745 | 0.533-1.040 | 0.031 | 0.691 | 0.494-0.967 |
| **Surgery about regional lymph nodes** | No surgery (ref) | 0.290 |  |  |  |  |  |
|  | Only biopsy | 0.964 | 0.000 | 2.881E+202 |  |  |  |
|  | Surgery and lymph node removed | 0.116 | 0.645 | 0.374-1.114 |  |  |  |

| **Metastatic including bone** | No(ref) | 0.742 |  |  |  |  |  |
| --- | --- | --- | --- | --- | --- | --- | --- |
|  | Yes | 0.742 | 1.061 | 0.747-1.505 |  |  |  |
| **Metastatic including liver** | No(ref) | 0.072 |  |  |  |  |  |
|  | Yes | 0.072 | 1.380 | 0.971-1.961 |  |  |  |
| **Metastatic including lung** | No(ref) | 0.722 |  |  |  |  |  |
|  | Yes | 0.722 | 1.063 | 0.759-1.488 |  |  |  |
| **Metastatic including liver** | No(ref) | 0.281 |  |  |  |  |  |
|  | Yes | 0.281 | 750 | 0.445-1.264 |  |  |  |
| **The number of metastatic sites** | One or two sites (ref) | 0.041 |  |  |  |  |  |
|  | Three or four sites | 0.019 | 1.936 | 1.116-3.357 |  |  |  |
|  | Distant metastatic sites can’t be assessed | 0.534 | 1.303 | 0.565-3.006 |  |  |  |

§. PUC: pure upper urinary tract urothelial cell carcinoma; UTVH: upper urinary tract tumors with variant histology
