# Supplementary material for: The role of surgery on primary site in metastatic upper urinary tract urothelial carcinoma and a nomogram for predicting the survival of patients with metastatic upper urinary tract urothelial carcinoma
Source: Cancer Med. 2021 Oct 14;10(22):8079–90. doi: 10.1002/cam4.4327 (PMC8607251; doi:10.1002/cam4.4327)
Supplement: Supplementary file 10 — Table S9 [file CAM4-10-8079-s002.docx]

Table S9 Univariable and multivariable Cox regression model analyses for overall survival of metastatic upper urinary tract urothelial carcinoma with N1/2/3 stage after PSM

| variables | level | univariable | | | multivariable | | |
| --- | --- | --- | --- | --- | --- | --- | --- |
|  |  | P value | HR | 95%CI | P value | HR | 95%CI |
| **Age at diagnosis (years)** | 70-79 | 0.012 |  |  |  |  |  |
|  | >79 | 0.012 | 1.404 | 1.077-1.830 |  |  |  |
| **Histologic type** | PUC(ref) | 0.330 |  |  |  |  |  |
|  | UTVH | 0.330 | 0.813 | 0.536-1.233 |  |  |  |
| **Grade** | I (ref) | 0.491 |  |  |  |  |  |
|  | II | 0.567 | 0.716 | 0.228-2.250 |  |  |  |
|  | III | 0.160 | 0.580 | 0.271-1.241 |  |  |  |
|  | IV | 0.869 | 1.023 | 0.780-1.342 |  |  |  |
| **T stage** | T1 (ref) | 0.018 |  |  | 0.035 |  |  |
|  | T2 | 0.476 | 1.288 | 0.642-2.584 | 0.465 | 1.301 | 0.643-2.633 |
|  | T3 | 0.124 | 1.467 | 0.900-2.391 | 0.014 | 1.901 | 1.136-3.178 |
|  | T4 | 0.024 | 1.741 | 1.076-2.817 | 0.018 | 1.865 | 1.113-3.124 |
|  | TX | 0.002 | 2.315 | 1.364-3.929 | 0.004 | 2.213 | 1.290-3.798 |
| **Radiotherapy** | No/unknown | 0.019 |  |  | 0.031 |  |  |
|  | Yes | 0.019 | 0.642 | 0.443-0.931 | 0.031 | 0.656 | 0.448-0.961 |
| **Chemotherapy** | No (ref) | <0.0001 |  |  | <0.0001 |  |  |
|  | Yes | <0.0001 | 0.397 | 0.305-0.519 | <0.0001 | 0.351 | 0.266-0.464 |
| **Surgery** | No (ref) | 0.017 |  |  | 0.027 |  |  |
|  | Yes | 0.017 | 0.799 | 0.618-1.033 | 0.027 | 0.702 | 0.512-0.961 |
| **Surgery about regional lymph nodes** | No surgery (ref) | 0.800 |  |  |  |  |  |
|  | Only biopsy | 0.633 | 1.179 | 0.600-2.315 |  |  |  |
|  | Surgery and lymph node removed | 0.696 | 0.947 | 0.722-1.242 |  |  |  |
| **Metastatic including bone** | No(ref) | 0.395 |  |  |  |  |  |
|  | Yes | 0.395 | 1.126 | 0.856-1.480 |  |  |  |
| **Metastatic including liver** | No(ref) | <0.0001 |  |  | <0.0001 |  |  |
|  | Yes | <0.0001 | 1.905 | 1.434-2.532 | <0.0001 | 1.901 | 1.419-2.547 |
| **Metastatic including lung** | No(ref) | 0.142 |  |  |  |  |  |
|  | Yes | 0.142 | 1.217 | 0.936-1.582 |  |  |  |
| **Metastatic including distant lymph node** | No(ref) | 0.498 |  |  |  |  |  |
|  | Yes | 0.498 | 0.910 | 0.693-1.196 |  |  |  |
| **The number of metastatic sites** | One or two sites (ref) | 0.007 |  |  |  |  |  |
|  | Three or four sites | 0.204 | 1.334 | 0.855-2.082 |  |  |  |
|  | Distant metastatic sites can’t be assessed | 0.003 | 2.339 | 1.323-4.137 |  |  |  |

§. PUC: pure upper urinary tract urothelial cell carcinoma; UTVH: upper urinary tract tumors with variant histology
