# Supplementary material for: The role of surgery on primary site in metastatic upper urinary tract urothelial carcinoma and a nomogram for predicting the survival of patients with metastatic upper urinary tract urothelial carcinoma
Source: Cancer Med. 2021 Oct 14;10(22):8079–90. doi: 10.1002/cam4.4327 (PMC8607251; doi:10.1002/cam4.4327)
Supplement: Supplementary file 11 — Table S10 [file CAM4-10-8079-s013.docx]

Table S10 Univariable and multivariable Cox regression model analyses for overall survival of metastatic upper urinary tract urothelial carcinoma with NX stage after PSM

| variables | level | univariable | | | multivariable | | |
| --- | --- | --- | --- | --- | --- | --- | --- |
|  |  | P value | HR | 95%CI | P value | HR | 95%CI |
| **Age at diagnosis (years)** | 70-79 | 0.409 |  |  |  |  |  |
|  | >79 | 0.409 | 1.278 | 0.713-2.291 |  |  |  |
| **Race** | Black(ref) | 0.140 |  |  |  |  |  |
|  | White | 0.988 | 0.988 | 0.191-5.109 |  |  |  |
|  | Other | 0.241 | 0.420 | 0.098-1.791 |  |  |  |
| **Histologic type** | PUC(ref) | 0.674 |  |  |  |  |  |
|  | UTVH | 0.674 | 1.203 | 0.508-2.852 |  |  |  |
| **Grade** | I (ref) | 0.562 |  |  |  |  |  |
|  | II | 0.204 | 0.205 | 0.018-2.363 |  |  |  |
|  | III | 0.195 | 0.255 | 0.032-2.012 |  |  |  |
|  | IV | 0.255 | 0.306 | 0.040-2.353 |  |  |  |
| **T stage** | T1 (ref) | 0.028 |  |  |  |  |  |
|  | T2 | 0.248 | 0.272 | 0.030-2.479 |  |  |  |
|  | T3 | 0.016 | 0.217 | 0.0630.748 |  |  |  |
|  | T4 | 0.066 | 0.338 | 0.106-1.075 |  |  |  |
|  | TX | 0.512 | 0.688 | 0.225-2.101 |  |  |  |
| **Radiotherapy** | No/unknown | 0.109 |  |  |  |  |  |
|  | Yes | 0.109 | 0.570 | 0.287-1.134 |  |  |  |
| **Chemotherapy** | No (ref) | <0.0001 |  |  | <0.0001 |  |  |
|  | Yes | <0.0001 | 0.231 | 0.113-0.472 | <0.0001 | 0.249 | 0.027-2.304 |
| **2.304Surgery** | No (ref) | 0.186 |  |  |  |  |  |
|  | Yes | 0.186 | 0.676 | 0.378-1.208 |  |  |  |
| **Metastatic including bone** | No(ref) | 0.579 |  |  |  |  |  |
|  | Yes | 0.579 | 0.847 | 0.471-1.523 |  |  |  |
| **Metastatic including liver** | No(ref) | 0.110 |  |  |  |  |  |
|  | Yes | 0.110 | 1.645 | 0.894-3.027 |  |  |  |
| **Metastatic including lung** | No(ref) | 0.970 |  |  |  |  |  |
|  | Yes | 0.970 | 0.990 | 0.561-1.747 |  |  |  |
| **Metastatic including distant lymph node** | No(ref) | 0.294 |  |  |  |  |  |
|  | Yes | 0.294 | 0.677 | 0.326-1.404 |  |  |  |
| **The number of metastatic sites** | One or two sites (ref) | 0.219 |  |  |  |  |  |
|  | Three or four sites | 0.297 | 0.658 | 0.300-1.445 |  |  |  |
|  | Distant metastatic sites can’t be assessed | 0.639 | 1.267 | 0.471-3.408 |  |  |  |

§. PUC: pure upper urinary tract urothelial cell carcinoma; UTVH: upper urinary tract tumors with variant histology
