# Supplementary material for: The role of surgery on primary site in metastatic upper urinary tract urothelial carcinoma and a nomogram for predicting the survival of patients with metastatic upper urinary tract urothelial carcinoma
Source: Cancer Med. 2021 Oct 14;10(22):8079–90. doi: 10.1002/cam4.4327 (PMC8607251; doi:10.1002/cam4.4327)
Supplement: Supplementary file 12 — Table S11 [file CAM4-10-8079-s005.docx]

Table S11 Univariable and multivariable Cox regression model analyses for overall survival of metastatic upper urinary tract urothelial carcinoma with liver metastasis after PSM

| variables | level | univariable | | | multivariable | | |
| --- | --- | --- | --- | --- | --- | --- | --- |
|  |  | P value | HR | 95%CI | P value | HR | 95%CI |
| **Age at diagnosis (years)** | 70-79 | 0.208 |  |  |  |  |  |
|  | >79 | 0.208 | 1.244 | 0.886-1.746 |  |  |  |
| **Race** | Black(ref) | 0.543 |  |  |  |  |  |
|  | White | 0.276 | 1.838 | 0.615-5.494 |  |  |  |
|  | Other | 0.443 | 1.433 | 0.572-3.590 |  |  |  |
| **Histologic type** | PUC(ref) | 0.689 |  |  |  |  |  |
|  | UTVH | 0.689 | 1.113 | 0.659-1.879 |  |  |  |
| **T stage** | T1 (ref) | 0.113 |  |  |  |  |  |
|  | T2 | 0.758 | 0.860 | 0.330-2.243 |  |  |  |
|  | T3 | 0.291 | 0.695 | 0.354-1.365 |  |  |  |
|  | T4 | 0.483 | 0.790 | 0.410-1.525 |  |  |  |
|  | TX | 0.415 | 1.345 | 0.660-2.742 |  |  |  |
| **N stage** | N0(ref) | 0.172 |  |  |  |  |  |
|  | N1/N2/N3 | 0.063 | 0.588 | 0.336-1.028 |  |  |  |
|  | NX | 0.195 | 0.702 | 0.411-1.198 |  |  |  |
| **Radiotherapy** | No/unknown | 0.423 |  |  |  |  |  |
|  | Yes | 0.423 | 0.791 | 0.445-1.405 |  |  |  |
| **Chemotherapy** | No (ref) | <0.0001 |  |  | <0.0001 |  |  |
|  | Yes | <0.0001 | 0.447 | 0.315-0.634 | <0.0001 | 0.4448 | 0.315-0.635 |
| **Surgery** | No (ref) | 0.032 |  |  | 0.033 |  |  |
|  | Yes | 0.032 | 0.691 | 0.494-0.968 | 0.033 | 0.693 | 0.495-0.971 |
| **Surgery about regional lymph nodes** | No surgery (ref) | 0.512 |  |  |  |  |  |
|  | Only biopsy | 0.788 | 0.825 | 0.203-3.352 |  |  |  |
|  | Surgery and lymph node removed | 0.254 | 0.798 | 0.525-1.186 |  |  |  |
| **Metastatic including bone** | No(ref) | 0.360 |  |  |  |  |  |
|  | Yes | 0.360 | 1.184 | 0.825-1.699 |  |  |  |
| **Metastatic including lung** | No(ref) | 0.679 |  |  |  |  |  |
|  | Yes | 0.679 | 0.930 | 0.659-1.312 |  |  |  |
| **Metastatic including distant lymph node** | No(ref) | 0.377 |  |  |  |  |  |
|  | Yes | 0.377 | 0.824 | 0.537-1.266 |  |  |  |
| **The number of metastatic sites** | One or two sites (ref) | 0.768 |  |  |  |  |  |
|  | Three or four sites | 0.768 | 1.059 | 0.724-1.548 |  |  |  |

§. PUC: pure upper urinary tract urothelial cell carcinoma; UTVH: upper urinary tract tumors with variant histology
