# Supplementary material for: The role of surgery on primary site in metastatic upper urinary tract urothelial carcinoma and a nomogram for predicting the survival of patients with metastatic upper urinary tract urothelial carcinoma
Source: Cancer Med. 2021 Oct 14;10(22):8079–90. doi: 10.1002/cam4.4327 (PMC8607251; doi:10.1002/cam4.4327)
Supplement: Supplementary file 13 — Table S12 [file CAM4-10-8079-s006.docx]

Table S12 Univariable and multivariable Cox regression model analyses for overall survival of metastatic upper urinary tract urothelial carcinoma with bone metastasis after PSM

| variables | level | univariable | | | multivariable | | |
| --- | --- | --- | --- | --- | --- | --- | --- |
|  |  | P value | HR | 95%CI | P value | HR | 95%CI |
| **Age at diagnosis (years)** | 70-79 | 0.034 |  |  |  |  |  |
|  | >79 | 0.034 | 1.432 | 4.028-1.994 |  |  |  |
| **Race** | Black(ref) | 0.626 |  |  |  |  |  |
|  | White | 0.414 | 0.677 | 0.265-1.827 |  |  |  |
|  | Other | 0.333 | 0.664 | 0.290-1.522 |  |  |  |
| **Histologic type** | PUC(ref) | 0.403 |  |  |  |  |  |
|  | UTVH | 0.403 | 0.804 | 0.483-1.339 |  |  |  |
| **Grade** | I (ref) | 0.024 |  |  |  |  |  |
|  | II | 0.002 | 0.037 | 0.004-0.312 |  |  |  |
|  | III | 0.006 | 0.055 | 0.007-0.443 |  |  |  |
|  | IV | 0.006 | 0.054 | 0.007-0.428 |  |  |  |
| **T stage** | T1 (ref) | 0.021 |  |  |  |  |  |
|  | T2 | 0.938 | 1.040 | 0.383-2.823 |  |  |  |
|  | T3 | 0.690 | 1.122 | 0.638-1.973 |  |  |  |
|  | T4 | 0.064 | 1.676 | 0.970-2.895 |  |  |  |
|  | TX | 0.009 | 2.156 | 1.213-3.833 |  |  |  |
| **N stage** | N0(ref) | 0.632 |  |  |  |  |  |
|  | N1/N2/N3 | 0.443 | 0.812 | 0.476-1.3833 |  |  |  |
|  | NX | 0.339 | 0.782 | 0.472-1.295 |  |  |  |
| **Radiotherapy** | No/unknown | 0.008 |  |  |  |  |  |
|  | Yes | 0.008 | 0.640 | 0.459-0.891 |  |  |  |
| **Chemotherapy** | No (ref) | <0.0001 |  |  | <0.0001 |  |  |
|  | Yes | <0.0001 | 0.421 | 0.300-0.590 | <0.0001 | 0.433 | 0.309-0.607 |
| **Surgery** | No (ref) | 0.008 |  |  | 0.020 |  |  |
|  | Yes | 0.008 | 0.640 | 0.459-0.891 | 0.020 | 0.675 | 0.485-0.940 |
| **Surgery about regional lymph nodes** | No surgery (ref) | 0.546 |  |  |  |  |  |
|  | Only biopsy | 0.593 | 1.368 | 0.433-4.320 |  |  |  |
|  | Surgery and lymph node removed | 0.358 | 0.827 | 0.552-1.239 |  |  |  |
| **Metastatic including liver** | No(ref) | 0.002 |  |  |  |  |  |
|  | Yes | 0.002 | 1.764 | 1.228-2.534 |  |  |  |
| **Metastatic including lung** | No(ref) | 0.097 |  |  |  |  |  |
|  | Yes | 0.097 | 1.341 | 0.948-1.896 |  |  |  |
| **Metastatic including distant lymph node** | No(ref) | 0.224 |  |  |  |  |  |
|  | Yes | 0.224 | 1.288 | 0.857-1.935 |  |  |  |
| **The number of metastatic sites** | One or two sites (ref) | 0.016 |  |  | 0.019 |  |  |
|  | Three or four sites | 0.016 | 1.598 | 1.092-2.339 | 0.019 | 1.574 | 1.077-2.300 |

§. PUC: pure upper urinary tract urothelial cell carcinoma; UTVH: upper urinary tract tumors with variant histology
