# Supplementary material for: The role of surgery on primary site in metastatic upper urinary tract urothelial carcinoma and a nomogram for predicting the survival of patients with metastatic upper urinary tract urothelial carcinoma
Source: Cancer Med. 2021 Oct 14;10(22):8079–90. doi: 10.1002/cam4.4327 (PMC8607251; doi:10.1002/cam4.4327)
Supplement: Supplementary file 14 — Table S13 [file CAM4-10-8079-s004.docx]

Table S13 Univariable and multivariable Cox regression model analyses for overall survival of metastatic upper urinary tract urothelial carcinoma with lung metastasis after PSM

| variables | level | univariable | | | multivariable | | |
| --- | --- | --- | --- | --- | --- | --- | --- |
|  |  | P value | HR | 95%CI | P value | HR | 95%CI |
| **Age at diagnosis (years)** | 70-79 | 0.883 |  |  |  |  |  |
|  | >79 | 0.883 | 1.023 | 0.753-1.391 |  |  |  |
| **Histologic type** | PUC(ref) | 0.047 |  |  |  |  |  |
|  | UTVH | 0.047 | 1.580 | 1.007-2.479 |  |  |  |
| **T stage** | T1 (ref) | 0.440 |  |  |  |  |  |
|  | T2 | 0.672 | 0.843 | 0.383-1.855 |  |  |  |
|  | T3 | 0.240 | 0.718 | 0.413-1.248 |  |  |  |
|  | T4 | 0.844 | 0.947 | 0.553-1.622 |  |  |  |
|  | TX | 0.889 | 1.042 | 0.583-1.863 |  |  |  |
| **N stage** | N0(ref) | 0.348 |  |  |  |  |  |
|  | N1/N2/N3 | 0.207 | 0.730 | 0.448-1.190 |  |  |  |
|  | NX | 0.152 | 0.712 | 0.448-1.133 |  |  |  |
| **Radiotherapy** | No/unknown | 0.106 |  |  |  |  |  |
|  | Yes | 0.106 | 0.646 | 0.380-1.098 |  |  |  |
| **Chemotherapy** | No (ref) | <0.0001 |  |  | <0.0001 |  |  |
|  | Yes | <0.0001 | 0.450 | 0.333-0.610 | <0.0001 | 0.453 | 0.335-0.612 |
| **Surgery** | No (ref) | 0.508 |  |  |  |  |  |
|  | Yes | 0.508 | 0.906 | 0.675-1.215 |  |  |  |
| **Surgery about regional lymph nodes** | No surgery (ref) | 0.951 |  |  |  |  |  |
|  | Only biopsy | 0.997 | 1.002 | 0.318-3.155 |  |  |  |
|  | Surgery and lymph node removed | 0.751 | 1.057 | 0.750-1.489 |  |  |  |
| **Metastatic including bone** | No(ref) | 0.192 |  |  |  |  |  |
|  | Yes | 0.192 | 1.244 | 0.896-1.727 |  |  |  |
| **Metastatic including liver** | No(ref) | 0.026 |  |  | 0.029 |  |  |
|  | Yes | 0.026 | 1.445 | 1.045-1.998 | 0.029 | 1.434 | 1.038-1.982 |
| **Metastatic including distant lymph node** | No(ref) | 0.493 |  |  |  |  |  |
|  | Yes | 0.493 | 0.890 | 0.639-1.241 |  |  |  |

§. PUC: pure upper urinary tract urothelial cell carcinoma; UTVH: upper urinary tract tumors with variant histology
