# Supplementary material for: The role of surgery on primary site in metastatic upper urinary tract urothelial carcinoma and a nomogram for predicting the survival of patients with metastatic upper urinary tract urothelial carcinoma
Source: Cancer Med. 2021 Oct 14;10(22):8079–90. doi: 10.1002/cam4.4327 (PMC8607251; doi:10.1002/cam4.4327)
Supplement: Supplementary file 15 — Table S14 [file CAM4-10-8079-s010.docx]

Table S14 Univariable and multivariable Cox regression model analyses for overall survival of metastatic upper urinary tract urothelial carcinoma with distant lymph node metastasis after PSM

| variables | level | univariable | | | multivariable | | |
| --- | --- | --- | --- | --- | --- | --- | --- |
|  |  | P value | HR | 95%CI | P value | HR | 95%CI |
| **Age at diagnosis (years)** | 70-79 | 0.865 |  |  |  |  |  |
|  | >79 | 0.865 | 1.035 | 0.695-1.542 |  |  |  |
| **Race** | Black(ref) | 0.160 |  |  |  |  |  |
|  | White | 0.905 | 1.067 | 0.371-3.063 |  |  |  |
|  | Other | 0.187 | 0.593 | 0.273-1.288 |  |  |  |
| **Histologic type** | PUC(ref) | 0.253 |  |  |  |  |  |
|  | UTVH | 0.253 | 1.466 | 0.761-2.823 |  |  |  |
| **Grade** | I (ref) | 0.319 |  |  |  |  |  |
|  | II | 0.199 | 2.974 | 0.565-15.661 |  |  |  |
|  | III | 0.491 | 1.655 | 0.394-6.955 |  |  |  |
|  | IV | 0.674 | 1.356 | 0.328-5.606 |  |  |  |
| **T stage** | T1 (ref) | 0.042 |  |  | 0.018 |  |  |
|  | T2 | 0.731 | 1.172 | 0.474-2.894 | 0.907 | 0.947 | 0.376-2.382 |
|  | T3 | 0.833 | 1.075 | 0.547-2.114 | 0.832 | 1.077 | 0.545-2.127 |
|  | T4 | 0.766 | 1.108 | 0.565-2.171 | 0508 | 1.257 | 0.638-2.476 |
|  | TX | 0.020 | 2.375 | 1.147-4.916 | 0.011 | 2.609 | 1.250-5.445 |
| **N stage** | N0(ref) | 0.808 |  |  |  |  |  |
|  | N1/N2/N3 | 0.516 | 0.762 | 0.336-1.728 |  |  |  |
|  | NX | 0.650 | 0.851 | 0.425-1.705 |  |  |  |
| **Radiotherapy** | No/unknown | 0.807 |  |  |  |  |  |
|  | Yes | 0.807 | 1.075 | 0.600-1.929 |  |  |  |
| **Chemotherapy** | No (ref) | <0.0001 |  |  | <0.0001 |  |  |
|  | Yes | <0.0001 | 0.472 | 0.315-0.707 | <0.0001 | 0.473 | 0.314-0.713 |
| **Surgery** | No (ref) | 0.012 |  |  |  |  |  |
|  | Yes | 0.012 | 0.606 | 0.410-0.897 |  |  |  |
| **Surgery about regional lymph nodes** | No surgery (ref) | 0.206 |  |  |  |  |  |
|  | Only biopsy | 0.639 | 0.786 | 0.287-2.153 |  |  |  |
|  | Surgery and lymph node removed | 0.079 | 0.658 | 0.412-1.050 |  |  |  |
| **Metastatic including bone** | No(ref) | 0.013 |  |  | 0.011 |  |  |
|  | Yes | 0.013 | 1.733 | 1.121-2.680 | 0.011 | 1.772 | 1.139-2.757 |
| **Metastatic including liver** | No(ref) | 0.048 |  |  |  |  |  |
|  | Yes | 0.048 | 1.576 | 1.004-2.474 |  |  |  |
| **Metastatic including lung** | No(ref) | 0.248 |  |  |  |  |  |
|  | Yes | 0.248 | 1.260 | 0.852-1.864 |  |  |  |

§. PUC: pure upper urinary tract urothelial cell carcinoma; UTVH: upper urinary tract tumors with variant histology
